# *In Situ* Temperature-Dependent Properties of Metal–Organic Framework ZIF-Coated ZnO Hybrid Structures: Structural and Advanced Spectroscopic Insights

**DOI:** 10.1021/acsomega.6c02238

**Published:** 2026-05-04

**Authors:** Rajat Nagpal, Masaya Sugihara, Cristian Lupan, Nicolae Magariu, Tim Tjardts, Nahomy Meling Lizarde, Thomas Strunskus, Justin Jetter, Haoyi Qiu, Barnika Chakraborty, Tayebeh Ameri, Eckhard Quandt, Rainer Adelung, Rob Ameloot, Oleg Lupan

**Affiliations:** 1 Center for Nanotechnology and Nanosensors, Department of Microelectronics and Biomedical Engineering, 187225 Faculty CIM, Technical University of Moldova, 168 Stefan cel Mare str., Chisinau MD-2004, Republic of Moldova; 2 Chair for Functional Nanomaterials, Department of Materials Science, Faculty of Engineering, 9179 Kiel University, Kaiserstraße 2, Kiel D-24143, Germany; 3 Centre for membrane separations, Adsorption, Catalysis, and Spectroscopy (cMACS), 26657 KU Leuven, Leuven 3001, Belgium; 4 Chair for Composite Materials, Department of Materials Science, Faculty of Engineering, 9179 Kiel University, Kaiserstraße 2, Kiel D-24143, Germany; 5 Chair of Inorganic Functional Nanomaterials, Department of Material Science, Faculty of Engineering, 9179 Kiel University, Kaiserstraße 2, Kiel D-24143, Germany; 6 Kiel Nano, Surface and Interface Science KiNSIS, 9179 Kiel University, Christian Albrechts-Platz 4, Kiel 24118, Germany

## Abstract

Advancements in material design, specifically the development of hybrids combining metal oxides (MO_*x*_) and zeolitic imidazolate frameworks (MOF/ZIFs), have revolutionized modern materials science by employing their synergistic effects and creating the hybrid interface MOF/MO_*x*_. This work systematically examines the structural evolution, defect engineering, and thermal stability of zeolitic imidazolate framework (ZIF-7, ZIF-8, ZIF-67, and ZIF-71)-coated ZnO and Cd-doped ZnO columnar structures. Comprehensive characterization was performed on all four studied ZIFs, including scanning electron microscopy revealing dodecahedral morphology of ZIF particles, and *in situ* temperature-dependent X-ray diffraction showing smaller coherently scattering regions compared to the sizes of the particles. SEM images indicate that these particles are polycrystalline and show a large contribution of surface-related domains. Thermolysis behavior and bond energy analysis reveal the role of Co–N bonds in thermal degradation of ZIF-67 at 250 °C compared to Zn–N bond of ZIF-8, which degrades at 325 °C, although Co–N has a higher bond energy compared to Zn–N, attributed to unsaturated coordination of Co with N, which leads to easy oxygenation of a Co–N bond. In ZIF-71 and ZIF-8, peak shifts are observed at higher angles by increasing the temperature, which would point to a negative thermal expansion. Using current–voltage characteristics, an inverted hysteresis was observed by employing forward and reverse voltage sweep, which may be characterized as capacitive hysteresis attributed to charge traps that slow down the return path. Gas-sensing studies revealed functional implications that ZIF-67-coated ZnO exhibited selective VOC sensing at 250 °C and enhanced hydrogen sensing at 300 °C, with structure–property correlations elucidated through defect analysis. Other hybrid materials, such as ZIF-7-coated ZnO, ZIF-8-coated ZnO, and ZIF-71-coated ZnO, elucidate better hydrogen, n-butanol, and 2-propanol sensing compared to ethanol and acetone. Our findings highlight the critical role of ZIF type and Cd doping in tuning the structural, thermal, and functional properties of ZnO-based composites, offering new perspectives for the rational design of advanced sensing materials based on the hybrid interface MOF/MO_*x*_.

## Graphical abstract

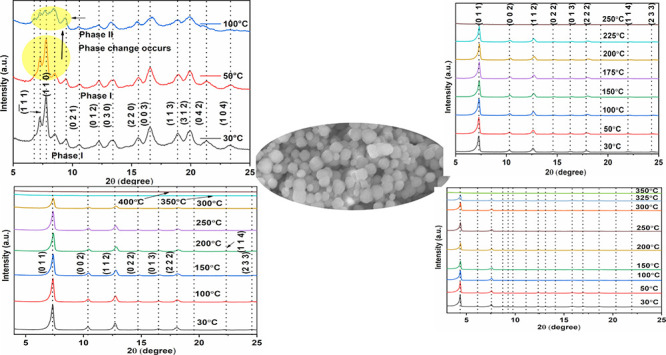

## 1 Introduction

Metal–organic frameworks (MOFs) are a special class of porous hybrid materials that consist of inorganic metal clusters and organic ligands connected by the coordination bonds.^1^ MOFs feature the modular design of extended networks, tunable pore sizes, and extremely high surface area.^2^ The modularity of organic linkers and metal nodes ensures precise tuning of the physical and chemical properties of MOFs for specific applications.^3^ Zeolitic imidazolate frameworks (ZIFs) are a subclass of MOFs with combined properties of zeolites and MOFs, including high surface area, microporous structure, and remarkable thermal and chemical stability.^1^

The stability of MOFs is crucial for developing hybrid materials suitable for a wide range of applications. Structural stability is particularly important for all types of ZIFs under varying operating conditions. *In situ* measurements have been widely employed to investigate the instantaneous changes in ZIFs at different operating temperatures or pressures. To assess the thermal stability of ZIFs, various characterizations such as *in situ* FTIR (Fourier transform infrared), among others, can be used. Xu et al.^4^ employed temperature-dependent *in situ* FTIR measurements on ZIF-8. Their results showed that below 200 °C, no significant changes in infrared (IR) peaks were observed. At higher temperatures, most IR peaks shifted to lower wavenumbers due to thermal expansion of the lattice. Flexible single bonds between C and H deform easily at elevated temperatures. Comparing *in situ* FTIR results with thermogravimetric analysis (TGA) revealed that framework decomposition involved a sharp weight loss in TGA analysis and the emergence of a C≡N peak at approximately 400 °C. This observation confirms the irreversible cleavage of Zn–N bonds within the framework structure.

Functional groups have a significant influence on the flexibility and adsorption properties of the ZIFs. For instance, methyl or -chloro functional group on the ZIF-8 framework enhance its flexibility, whereas the presence of a -bromo functional group reduces flexibility during N_2_ adsorption.^5^ ZIF-7 possesses a flexible network that undergoes a phase transition from a narrow-pore phase to a large-pore phase in the presence of penetrating solvents such as alcohols, a phenomenon known as the gate-opening effect. *In situ* pressure-dependent measurements demonstrate its structural transformation to a triclinic phase upon increasing pressure to 1 GPa, with further pressure increase to 5.65 GPa leading to amorphization.^6^

Temperature-dependent *in situ* FTIR analysis reveals deformation of the ZIF-8 structure at 350 °C, with structural degradation occurring at 400 °C. On the other hand, ZIF-67 shows no deformation but undergoes structural degradation at 325 °C. These findings were corroborated by TGA/DTG analysis.^7^ The temperature dependence of structural degradation may vary depending on the synthesis conditions. Moreover, Gadipelli et al. demonstrated that metal–nitrogen bonds in ZIF-8 and ZIF-67 break more easily than the organic linkers.^8^

ZnO is a versatile *n*-type semiconductor oxide that can be employed in a wide range of applications, including photodetection,^9^ solar energy harvesting,^10^ nanoelectronics,^11^ sensing of VOCs,^12,13^ components in electrical batteries,^14^ and the detection of other pollutants.^15^ ZnO is a direct bandgap semiconductor that possesses good electrical and thermal stability.^12^ It can adsorb molecular oxygen species on its surface, which capture electrons from the conduction band of ZnO.^16^ Consequently, ZnO is an appropriate candidate for gas-sensing applications. However, bare ZnO exhibits a magnificent sensitivity to a variety of gases simultaneously, which can limit its selectivity and real applications. The desired sensing attributes of ZnO can be improved by engineering its physical and chemical properties, typically achieved by incorporating various dopants or by adding a sheath layer to ZnO to enhance selectivity toward target analytes.^17,18^ Among various dopants, Cd incorporation into ZnO can significantly alter its electrical and chemical properties depending on the doping concentration.^55,124^ At the same time, by doping ZnO with Cd impurities and coating it with a poly­(ethylene glycol) dimethacrylate (PEGDMA) polymer layer, a dual-mode sensor was developed. It detects battery-related compounds at low operating temperatures and VOCs at high temperatures, featuring short response and recovery times.^19^ Coating Ag_2_S nanodots over ZnO doped with Cd impurities allowed one to obtain a sensor that detects UV radiation.^20^ Cd doping modifies the ZnO lattice, affecting the band gap and consequently the electrical conductivity.^55,124^ This effect is attributed to an increase in carrier concentration and the larger ionic radius of Cd^2+^ (0.92 Å) compared to Zn^2+^ (0.74 Å).^21^ The lattice expansion induced by Cd incorporation narrows the band gap, thereby enhancing the electrical conductivity.^22^

The transport of gases with similar reactivity can be modulated by applying a MOF sheath layer on the ZnO film.^23^ Developing hybrid materials by constructing an MOF layer on metal oxide films is an effective material design strategy that combines the high sensitivity of metal oxides with the selective functionality of MOFs. For example, Huang et al. demonstrated enhanced ethanol sensing performance of the ZIF-8-coated ZnO nanorods compared to bare ZnO nanorods.^24^ This improvement was attributed not only to the molecular sieving effect, resulting from the smaller pore size relative to ethanol molecules, but also to Zn^2+^-related defects that contributed to the enhancement in the sensing performance.

ZIF-8 and ZIF-67 share the same sodalite topology, and both use 2-methylimidzolate as the linker, but they differ in their metal nodes: Zn^2+^ in ZIF-8 and Co^2+^ in ZIF-67.^25,26^ Both are commonly employed in gas-sensing applications. Selective sensing of hydrogen molecules through ZIF-8 has been attributed to the molecular sieving effect,^27^ while ZIF-8 also shows stronger affinity toward higher-chain alcohols.^25,28^ Matatagui et al.^29^ demonstrated chemoresistive sensing based on ZIF-67, which exhibited a similar response (∼1.5) to 90 ppm of ethanol and hydrogen, indicating low sensitivity and poor selectivity. However, by combining ZIF-8 and ZIF-67 structures, a synergistic effect was observed, resulting in a notable increase in sensitivity, particularly toward toluene (10 ppm) and hydrogen (10 ppm), by factors of 5.6 and 4.8, respectively. A significant enhancement in selectivity was also observed for toluene (∼4-fold) and hydrogen (∼3-fold) compared with other tested analytes. Moreover, other ZIFs have also been investigated. The effect of the organic linker in ZIFs on the interaction mechanism with analytes can be understood by considering ZIFs such as ZIF-7 and ZIF-71, which have same metal nodes (Zn^2+^) but different organic linkers—benzimidazole and 4,5-dicholoroimidazole, respectively. For instance, Keyvanloo et al.^30^ studied the diffusion of guest molecules through different channels in ZIF-7 and demonstrated that the diffusion behavior is governed by the kinetic diameters of the molecules. Hydrogen, having the smallest kinetic diameter, diffused readily through the ZIF-7 pores, whereas VOCs with larger molecular sizes and greater steric hindrance experienced restricted diffusion. In a separate study, hydrogen adsorption on ZnO@ZIF-71@Ag revealed preferential adsorption at the Zn^2+^ sites. The distorted structure of ZIF-71 further contributed to selective molecular sieving by effectively blocking the passage of larger molecules.^31^ Sequential detection of a common set of test gases under identical conditions using individual sensors in an array has been reported to produce distinguishable response patterns, suggesting a potential approach for differentiating among alcohols and other gases.^32^

Microdrop-casting is a straightforward, simple, and cost-effective technique for the deposition of MOFs between interdigitated electrode (IDE) fingers.^33^ Controlled, repetitive drop-casting of an MOF suspension between the IDE fingers on metal oxide layers can combine the high sensitivity provided by metal oxides with the selectivity offered by MOFs. Campbell et al.^34^ reported the fabrication of a MOF-based sensor array using the drop-casting technique; however, the observed sensing response was relatively low. Following this approach, we fabricated hybrid materials consisting of a metal oxide film coated with a MOF layer via a microdrop-casting technique.

Numerous studies have explored the material properties of various ZIFs synthesized through different methods.^35–41^ However, to the best of our knowledge, no study has systematically compared suspensions of different ZIFs prepared at the same concentration and under identical synthesis conditions. Furthermore, only a limited number of reports have demonstrated the use of simple and cost-effective deposition techniques—such as microdrop-casting or spin-coating—for integrating different ZIFs onto metal oxide films grown by a cost-effective chemical approach as synthesis from chemical solutions (SCSs).^42,43^

The current study aims to design ZIF/ZnO-based hybrids using synthesis from chemical solutions (SCSs) and room temperature microdrop-casting. These techniques offer lower energy consumption and production costs than conventional methods, facilitating the integration of hybrid sensors into wearable devices. So far, systematic investigations of the physicochemical properties of these ZIFs under identical conditions have not been reported. We conducted a comprehensive investigation into the material properties of various ZIFs, including crystallite size, *in situ* temperature-dependent X-ray diffraction (XRD), Raman spectroscopy, defect analysis, chemical surface composition, surface chemistry by XPS, and gas-sensing performance at different temperatures. To ensure a clear and direct comparison, *in situ* temperature-dependent XRD was conducted under identical conditions for a variety of ZIFs (ZIF-67, ZIF-7, ZIF-71, and ZIF-8). This approach establishes the fundamental mechanisms of thermal degradation and crystal-phase transition across a series of samples. The investigation identified thermal stability intervals and the transformation of ZIFs into their corresponding metal oxides (ZnO or Co_3_O_4_), ultimately confirming their operational safety in sensory devices. Furthermore, we explored the Cd-doping effect on ZIF-coated ZnO-based hybrid materials. Additionally, we determined the activation energies of all the studied samples, elucidating the thermally activated conduction pathways. These findings provide a physical understanding of charge transport and molecular interactions at the hybrid interface MOF/MO_*x*_. Specifically, this work clarifies how charge transfer and the modulation of the potential barrier at the heterostructure interface contribute to the sensor signal, establishing a predictive framework for optimizing high-performance sensor technology.

## 2 Experimental Section

### 2.1 Materials Preparation

ZnO films were deposited on glass substrates (12 × 14 × 1 mm) using a simple and cost-efficient synthesis from the chemical solution (SCS) approach.^42,43^ Prior to deposition, glass substrates were cleaned in a dilute HCl (20%) solution for 10 min, followed by rinsing with deionized (DI) water with an electrical resistivity of 18.2 MΩ cm. Subsequently, the substrates were ultrasonically cleaned in a 1:1 mixture of ethanol and acetone for 5 min, then with DI water to remove organic residues and any remaining species from the surface and then dried using a N_2_ gas flux. After this treatment, the substrates exhibited hydrophilic properties. To ensure good film coverage or uniform deposition, the substrates were sensitized by dipping into a SnCl_2_·2H_2_O/HCl solution.^42,43^

The cationic precursor solution was prepared by using Zn­(SO_4_)·7H_2_O (99%, Sigma-Aldrich) and NaOH (99%, Sigma-Aldrich). The solution was diluted with DI water to obtain Zn concentrations ranging from 0.25 to 0.75 M, as previously reported.^42^ The cationic solution was maintained at room temperature, while the anionic aqueous solution was kept at 95–98 °C during deposition.

The ZnO growth cycle involved four dip-coating steps performed using an articulated robot to minimize human errors in the dipping times. The initial step consisted of immersing the substrates in the cationic precursor solution for 3 s. This was followed by rinsing the substrate in DI water to remove loosely attached cationic ions, ensuring uniform deposition. For the growth of Cd-doped samples, 0.5 mg of CdSO_4_ (≥99%, Sigma-Aldrich) was added to 500 mL of DI water, resulting in a Cd concentration of 5.46 μM for samples labeled M0 to M4. Next, the substrates were immersed in an anionic solution at 95 to 98 °C for 3 s, where ZnO:Cd formation occurred at the interface. Finally, the substrates were dipped again in DI water to remove excess unreacted species. These deposition cycles were repeated multiple times to achieve the desired film thickness, depending on the growth kinetics, as reported previously.^43^ These films were then thermally annealed at 650 °C for 2 h in air.

The four types of ZIFs (ZIF-67, ZIF-7, ZIF-71, and ZIF-8) were synthesized, where all of the reactions were carried out at room temperature. ZIF-67 was synthesized based on the previously reported method.^44^ Briefly, 1.35 g (4.6 mmol) of cobalt nitrate hexahydrate (≥98%, Sigma-Aldrich) and 16.35 g (199.1 mmol) of 2-methylimidazole (99%, Sigma-Aldrich) were dissolved separately in 9 and 60 mL of Milli-Q water, respectively. The latter linker solution was added to the former cobalt solution under stirring. After 6 h of stirring, purple solids were separated from the solvent by centrifugation and subsequently washed two times with Milli-Q water and two times with methanol (≥99.9%, Fisher Chemical). For ZIF-7 synthesis, zinc and linker solutions were prepared separately by dissolving 892.47 mg (3 mmol) of zinc nitrate hexahydrate (99%, Fisher Chemical) in 60 mL of *N*,*N*-dimethylformamide (DMF, ≥99.5%, Fisher Chemical) and 1417.68 mg (12 mmol) of benzimidazole (≥99%, Sigma-Aldrich) in 60 mL of methanol (≥99.9%, Fisher Chemical). After complete dissolution, the linker solution was added to the zinc solution, and the mixture was stirred for 1 min. After 24 h, white solids were separated from the solvent by centrifugation and subsequently washed three times with methanol. ZIF-71 was synthesized according to the method reported previously.^45^ Briefly, 351.22 mg (1.6 mmol) of zinc acetate dihydrate (≥99%, Sigma-Aldrich) was dissolved in 40 mL of methanol (≥99.9%, Fisher Chemical) and 876.54 mg (6.4 mmol) of 4,5-dichloroimidazole (>97%, Tokyo Chemical Industry Co., Ltd.) was dissolved in the mixture of methanol and DMF (methanol/DMF = 39.64/0.36, v/v). Later, the linker solution was added to the former zinc solution under stirring. After 4 h of stirring, white solids were separated from the solvent by centrifugation and subsequently washed three times with methanol. For ZIF-8 synthesis, zinc and linker solutions were prepared separately by dissolving 892.47 mg (3 mmol) of zinc nitrate hexahydrate (99%, Fisher Chemical) in 60 mL of methanol and 985.20 mg (12 mmol) of 2-methylimidazole (99%, Sigma-Aldrich) in 60 mL of methanol (99.9%, Fisher Chemical). After complete dissolution, the linker solution was added to the zinc solution, and the mixture was stirred for 1 min. After 24 h, white solids were separated from the solvent by centrifugation and subsequently washed three times with methanol. Afterward, the resultant ZIF particles were kept in methanol in dark. The sequence of sensors was developed using 10 different sensor types as tabulated in Table 1.

**Table 1:** Codification Scheme for All the 10 Sample Sets Included, with Corresponding Sample Names

sample name	sample codification
ZnO	0A
ZIF-67-coated ZnO	1A
ZIF-7-coated ZnO	2A
ZIF-71-coated ZnO	3A
ZIF-8-coated ZnO	4A
Cd-doped-ZnO (ZnO:Cd)	M0
ZIF-67-coated Cd-doped ZnO	M1
ZIF-7-coated Cd-doped ZnO	M2
ZIF-71-coated Cd-doped ZnO	M3
ZIF-8-coated Cd-doped ZnO	M4

All ZIF dispersions were prepared in methanol at a concentration of 5 mg/mL, with particle sizes of approximately 70 nm for ZIF-8, 200 nm for ZIF-7 and ZIF-67, and 500 to 700 nm (broad-range distribution) for ZIF-71. Before ZIF deposition, Au IDEs with a thickness of 170 nm were patterned onto the ZnO or Cd-doped ZnO samples using a metal mask in a meander configuration, maintaining a 1 mm gap between adjacent electrodes, as previously reported.^46^ The ZIF dispersions were ultrasonicated for 15 min to ensure homogeneity and then drop-casted onto the ZnO and Cd-doped ZnO samples: 80 μL was applied to each ZnO-based sample and 50 μL to each Cd-doped ZnO sample. Sensors were labeled according to their composition, as described above. A schematic of the device structure with ZIF particles on a ZnO or ZnO:Cd film—supported by a glass substrate with gold IDEs for electrical contacts—is illustrated in Figure S1.

### 2.2 Characterization

Morphological analysis of all samples was conducted using a Carl Zeiss scanning electron microscope (SEM) operated at 3–7 kV and 10 μA. Compositional analysis was performed by energy-dispersive X-ray spectroscopy (EDX) using a Zeiss Gemini Ultra55 Plus system attached to the SEM.

XRD measurements were carried out using a Rigaku SmartLab diffractometer (Japan) equipped with a high-power X-ray source of 9 kW (45 kV, 200 mA) and Cu K_α1_ radiation (1.54 Å) in a parallel-beam (PB) configuration. XRD patterns were recorded over a 2θ range of 2 to 80° with a step size of 0.05°. *In situ* XRD measurements were performed on all 10 sample sets to examine crystal-phase changes and degradation temperatures of the tested ZIFs using an Anton-Paar DHS 1100 temperature stage. *In situ* XRD patterns were collected over a 2θ range of 4 to 40° with a step size of 0.04°. All *in situ* measurements were taken at a scan speed of approximately 11.48° per minute. Each ZIF sample was exposed to X-rays for approximately 3.14 min at each temperature. High-resolution diffraction data were acquired using the HyPix-3000 hybrid photon counting (HPC) one-dimensional detector in line mode, which offers enhanced sensitivity and low noise performance.

Micro-Raman measurements were performed using a WITec alpha 300 RA system to investigate the structural, vibrational, and chemical information on the samples. The excitation source for most samples was a 532 nm green laser-line from a Nd:YAG laser (8 mW). However, for two ZIF-67-based sample sets, a 633 nm red laser at low intensity was used to avoid sample damage observed at 532 nm. The inelastically scattered light was analyzed by a triple-grating spectrometer equipped with a charge-coupled device (CCD) detector optimized for a blazing wavelength of 500 nm. The groove density of the gratings used to disperse Raman-scattered light varied, depending on the desired spectral range. High-density gratings (1800 grooves/mm) provided high spectral resolution but limited spectral range (∼900 cm^–1^) due to detector size constraints. Lower-density gratings (1200 and 600 grooves/mm) enabled wider spectral ranges of approximately 1400–1600 and 3500 cm^–1^, respectively.

To investigate the chemical surface composition and surface chemistry of different ZIF-coated Cd-doped ZnO samples, X-ray photoelectron spectroscopy (XPS, XPS UHV system from PREVAC Sp. z o. o., Al-anode, 300 W) was utilized. Survey scans were performed with 3 iterations and a pass energy of 200 eV while high-resolution scans were performed at 20 iterations and a pass energy of 50 eV. For the analysis of the XPS spectra, the software CasaXPS (version 2.3.25) was utilized. Here, the Shirley algorithm was used for estimating the background of each high-resolution spectrum. Compositional analysis was performed by numerical integration of the area between the background line and the corresponding high-resolution spectrum data, considering the peak specific relative sensitivity factors. The charge correction was implemented similar to the charge correction process reported in previous studies on ZIF-coated CuO.^1,47^ The binding energy (*E* *_b_*) of the central transition metal cation 2p_3/2_ peak was set to a reference value, and all other spectra from the same sample set were shifted accordingly. For the ZIF-7, ZIF-71, and ZIF-8 samples containing Zn, the fit of the Zn 2p_3/2_ peak of each sample was set to 1021.7 eV according to the reported literature.^48^ For the ZIF-67 sample, which possesses a Co^2+^ ion as the metal coordination center, the Co 2p_3/2_ was set to 781.2 eV according to the reported literature.^49^

Thermogravimetric analysis (TGA) was performed on bulk ZIF powder samples using a Jupiter STA 449 F3 thermogravimetric analyzer under a synthetic air flow of 50 mL/min. The samples were heated from 25 to 800 °C with a heating rate of 5 °C/min.

### 2.3 Gas-Sensing Experiments

For electrical and gas-sensing measurements, a two-point probe configuration connected to a Keithley 2400 sourcemeter was used. The system was interfaced with a LabView (National Instruments) platform to enable real-time acquisition and monitoring of the sensor’s electrical signals (electrical current or electrical resistance).

All sample sets were tested for gas-sensing performance against a range of analytes, including VOCs (acetone, n-butanol, ethanol, and 2-propanol) and hydrogen gas, each at a concentration of 100 ppm diluted in air.^42,43,124^ Measurements were conducted at various operating temperatures ranging from 20 to 300 °C. The measurement setup consisted of a quartz closed chamber connected to the gas sources via a pipe network with a diameter of 5 mm. These gases were diluted with air and introduced into a gas mixer using separate mass flow controllers (MFCs), as reported previously.^42,43,124^

The final concentration of the test gases after mixing with air was calculated using the equation:

$$C_{\mathrm{mix}}=C_{i}\times \frac{F_{\mathrm{gas}}}{F_{\mathrm{mix}}}$$ 1

where *C* *_i_* is the initial concentration of test gas introduced into the mixer, *F*_gas_ is the gas flow rate, *C*_mix_ is the final concentration of the test gas after mixing, and *F*_mix_ is the final flow rate of the mixed gas for tests.

The target gas mixture was introduced into the test chamber at a controlled flow rate of 200 standard cubic centimeters per minute (sccm) using precalibrated MFCs. Samples were placed on a heating stage and the operating temperature was precisely controlled and monitored via a microcontroller-based feedback system.

Gas-sensing measurements were performed under a fixed relative humidity (RH) of 10%. The air was introduced directly into the gas mixer without additional humidification. The RH in the gas mixer was continuously monitored using a hygrometer, as reported previously.^1,47^

The sensing response (*S*) of each sensor was calculated using the formula:^13^

$$S=\frac{R_{a}-R_{g}}{R_{g}}\times 100\%$$ 2

where *R* *_a_* is the baseline electrical resistance of the sample in air and *R* *_g_* is the electrical resistance of the sample in the presence of the test gas.

Response and recovery times were determined from the dynamic response curves, which indicate the duration a sensor takes to transition from 10 to 90% and from 90 to 10% of the maximum response value, respectively. The error for the determined response and recovery times is averaged out in the range of ± 0.5 s.^1,47^

## 3 Results and Discussion

### 3.1 Morphological and Structural Characterization of Individual Sensors

The surface morphology of all the samples was investigated using SEM. Following the microdrop-casting of four different ZIFs (ZIF-67, ZIF-7, ZIF-71, and ZIF-8) onto the ZnO and Cd-doped ZnO films, the ZIF particles were uniformly ditributed. The lower magnification images of ZIF-67 and ZIF-7 particles on ZnO films are shown in Figure 1a,c, respectively. It shows dodecahedral morphology with particle size of approximately 200 and 200 nm, respectively, which is estimated by scaling on SEM images of ZIF particles. Similarly, higher-magnification images for ZIF-67 and ZIF-7 particles are shown in Figure 1b,d, respectively. Figure S2­(a,c) shows the SEM micrographs at lower magnifications for ZnO and Cd-doped ZnO films, respectively. The compactly denser and uniformly distributed interconnected grains can be observed due to thermal annealing effect. Moreover, the SEM micrographs at higher magnifications for ZnO and Cd-doped ZnO films are shown in Figure S2­(b,d), respectively. It reveals the columnar morphology of ZnO films, and after Cd doping, additional columns growing out from the ZnO columns can be observed.

**Figure 1:**
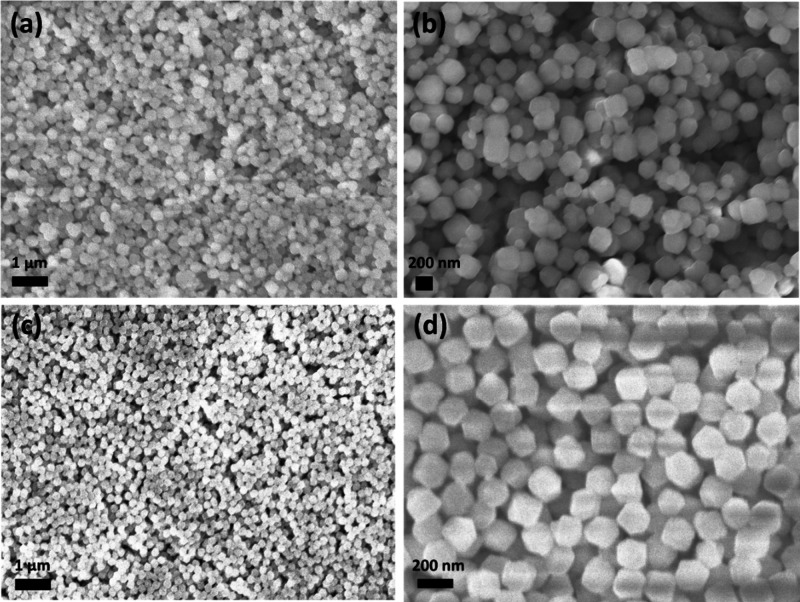
SEM images of ZIF-coated ZnO films at lower magnifications at 1 μm: (a) ZIF-67 coated on ZnO films and (c) ZIF-7 particles coated on ZnO films; and at higher magnifications at 200 nm: (b) ZIF-67 coated on ZnO films and (d) ZIF-7 particles coated on ZnO films.

Moreover, Figure 2a,c shows the uniform distribution of ZIF-71 and ZIF-8 particles with dodecahedral morphology and particle sizes of 500 to 700 nm (broad size distribution) and 70 nm, respectively. Furthermore, SEM micrographs of ZIF-71 and ZIF-8 particles on ZnO films at higher magnifications are shown in Figure 2b,d, respectively. These images reveal almost complete coverage of the ZnO films with ZIF particles for all studied ZIFs. The SEM images for ZIF-71 appear slightly blurry due to the charging effect of the insulating ZIF-71 particles. The presence of the Cd dopant in the Cd-doped ZnO films was confirmed via elemental mapping and compositional analysis using EDX. The analysis revealed Cd with a composition of 0.18 at. %.

**Figure 2:**
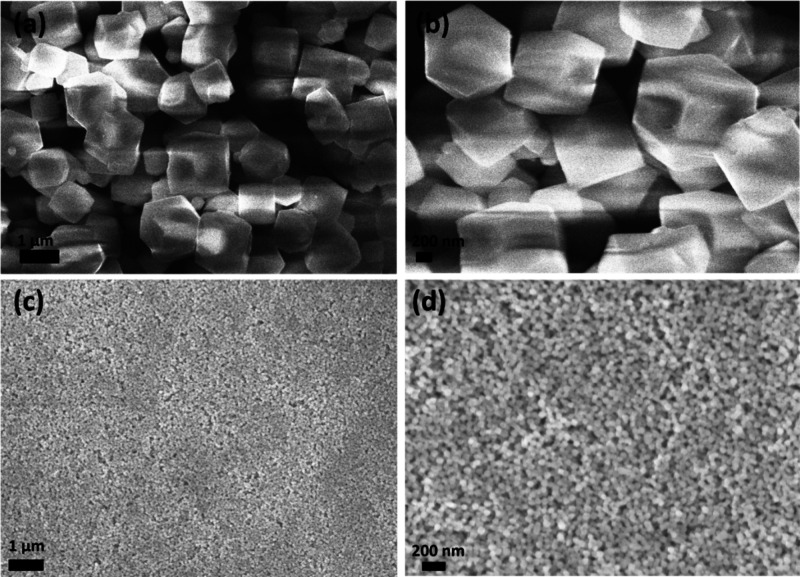
SEM images of ZIF-coated ZnO films at lower magnifications at 1 μm: (a) ZIF-71 coated on ZnO films and (c) ZIF-8 particles coated on ZnO film sample sets and at higher magnifications at 200 nm: (b) ZIF-71 coated on ZnO films and (d) ZIF-8 particles coated on ZnO films.

Figure 3a,b shows the XRD patterns of two sample series: 0A, 1A, 2A, 3A, and 4A and M0, M1, M2, M3, and M4, respectively. Distinct diffraction peaks were observed for both the ZIF-coated ZnO samples series (1A–4A) and the reference sample (0A) in Figure 3a, indicating good crystallinity. The XRD pattern for sample 2A in Figure 3a shows relatively low-intensity peaks compared with the other samples. However, when plotted independently, the XRD peaks corresponding to ZIF-7 are clearly visible, as shown in Figure S3. Similarly, the diffraction patterns for Cd-doped ZnO samples coated with ZIFs (M1-M4), along with the reference sample (M0), exhibited comparable diffraction features, as shown in Figure 3b. Characteristic peaks corresponding to ZIF-7,^50^ ZIF-8,^51^ ZIF-67,^52^ and ZIF-71 ^53^ are clearly visible and have been marked in both figures, confirming the presence of the respective frameworks. Overall, the XRD results verify the successful crystallization of all of the ZIF-based composites and hybrid sample sets. The dominant reflection for ZnO was observed at 34.43 ° corresponding to the (0 0 2) plane, indicating preferential growth along the *c*-axis, perpendicular to the substrate.

**Figure 3:**
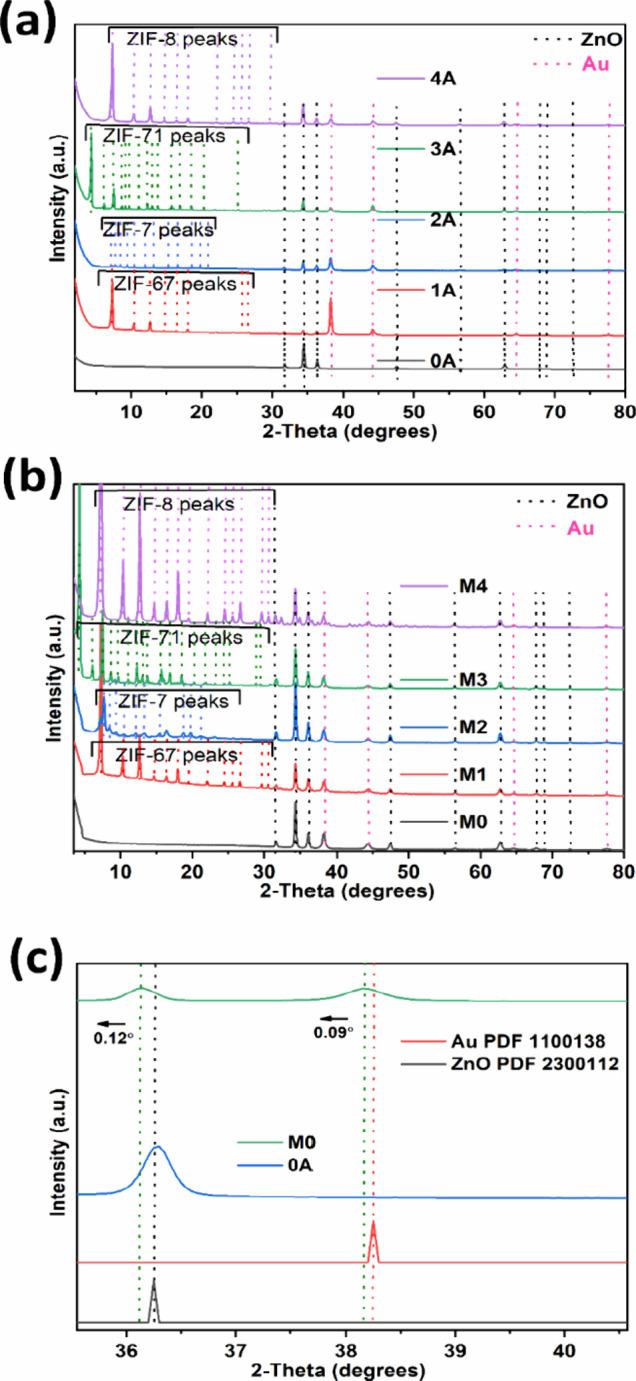
XRD patterns of (a) 0A (ZnO), 1A (ZIF-67-coated ZnO), 2A (ZIF-7-coated ZnO), 3A (ZIF-71-coated ZnO), and 4A (ZIF-8-coated ZnO) samples; (b) M0 to M4, and (c) comparison of 0A (ZnO) and M0 (ZnO:Cd) sample sets to show the crystallographic peak shift due to Cd doping.

Figure 3c shows the XRD patterns of uncoated ZnO and Cd-doped ZnO samples. These patterns match the standard diffraction reflections of ZnO (PDF 2300112), corresponding to the hexagonal Wurtzite structure. Comparison between 0A and M0 samples revealed a shift of approximately 0.12 ° to lower 2θ values in the (1 0 1) reflection of 0A, as shown in Figure 3c.

IDEs of Au were used for electrical contacts, and their corresponding reflections matched those of Au (PDF 1100138), with space group Fm_–3_m (2 2 5). A similar shift of about 0.09 ° to lower 2θ was observed in the Au (1 1 1) reflection after Cd doping, also shown in Figure 3c. This shift is consistent with previous reports on structural effects of Cd doping in ZnO.^21,54,55^ The lattice expansion can be attributed to the substitution of Zn^2+^ (ionic radius ∼0.74 Å) with larger Cd^2+^ ions (∼0.92 Å) at crystallographic positions.^21^ The presence of Cd dopant is already confirmed by EDX compositional analysis, though it is also supported by a shift in XRD reflection.

XRD patterns recorded at room temperature were used to estimate the crystallite size (*D*) of each ZIF, using the Scherrer equation:^56^

$$D=\frac{K\times \lambda }{\beta \times \cos\theta }$$ 3

where *K* (∼0.94) is the shape factor, λ (0.15406 nm) is the wavelength of the X-rays, β is the full width at half-maximum (fwhm in radians), and θ is the Bragg’s angle (in radians).

For all of the ZIF particles, the shape factor was taken as ∼0.94, which is typically used for cubic-shaped particles. The reflection with the highest intensity of each sample was selected for peak fitting to determine the fwhm. A Voigt function was chosen for peak fitting using the Orthogonal Distance Regression (Pro) iteration algorithm. For calculating fwhm (β in radians), the two-step approximation empirical formula for the Voigt profile was used:^57^

$$\beta \approx 0.5346\times w_{L}+\sqrt{0.2166\times w_{L}^{2}+w_{G}^{2}}$$ 4

where *w* *_L_* is the Lorentzian width and *w* *_G_* is the Gaussian width. The error for β profile is ± 0.02%.^57^

This empirical formula is derived from a more general formula for fitting the Voigt profile:^58^

$$\beta \approx w_{G}\left[1+0.5d+\left(1-e^{-1.65\times d}\right)\times \left(1.36603-0.47719d+0.11116\times d^{2}\right)\right]$$ 5

where *d = w* *_L_* */w* *_G_*. The error of this formula is ±0.01%.^58^

From all of these parameters, the crystallite sizes of all ZIF particles, calculated using eq 3, are also summarized in Table 2.

**Table 2:** Values of All the Parameters Used in the Scherrer Equation

name of the ZIF	reflection with the highest intensity	w_L_ (radians)	w_G_ (radians)	β (radians)	θ (radians)	*D* (nm)
ZIF-7	(1 1 0)	0.002635	0.002293	0.004069	0.068067	∼35.67
ZIF-67	(0 1 1)	0.001948	0.002211	0.003431	0.063873	∼42.30
ZIF-71	(1 1 0)	0.001402	0.001447	0.002339	0.038034	∼61.97
ZIF-8	(0 1 1)	0.002248	0.002450	0.003870	0.064232	∼37.50

The crystallite sizes calculated using eq 3 compared with the crystallite sizes of all ZIF particles is estimated to be smaller than the particle sizes observed in SEM images. Since the Scherrer equation accounts only for the coherently scattering regions of a particle and does not include surface-related domains, this discrepancy indicates that the particles observed in the SEM images are likely polycrystalline in nature.^59^ This broadening (β) is attributed to the small size of the coherently scattering monocrystalline regions.^60^ The observed particle sizes from SEM images for ZIF-71 are greater than 100 nm, which may give unreliable crystallite size results using the Scherrer equation,^60,61^ especially for the ZIF-71 with a particle size in the range of 500–700 nm. Thus, calculations of the crystallite size dimensions are not valid in this case. For the other ZIFs (ZIF-67, ZIF-7, and ZIF-8), the particle size is close to 100 nm, which shows minimal error.^59,61^

Using eq 4, the fwhm (β, in radians) of respective highest intensity of XRD peaks for all the ZIF particles was calculated as follows: e.g., for the ZIF-7 particles, the highest-intensity reflection (1 1 0) is observed at θ = 0.068067 radians, resulting in β = 0.004069 radians. The values of *w* *_L_* and *w* *_G_* were found to be 0.002635 and 0.002293 radians, respectively. Similarly, the corresponding values of *w* *_L_*, *w* *_G_*, *β,* and θ for all samples are tabulated below, with “radians” as the unit of the angle.

### 3.2 Thermal Stability Test of ZIFs Using *In Situ* XRD Measurement

To understand the thermal effect on the crystal structure of all ZIFs, *in situ* XRD measurements were carried out on all ZIF samples microdrop-casted on Si wafers for reference, as shown in Figure 4a–f.

**Figure 4:**
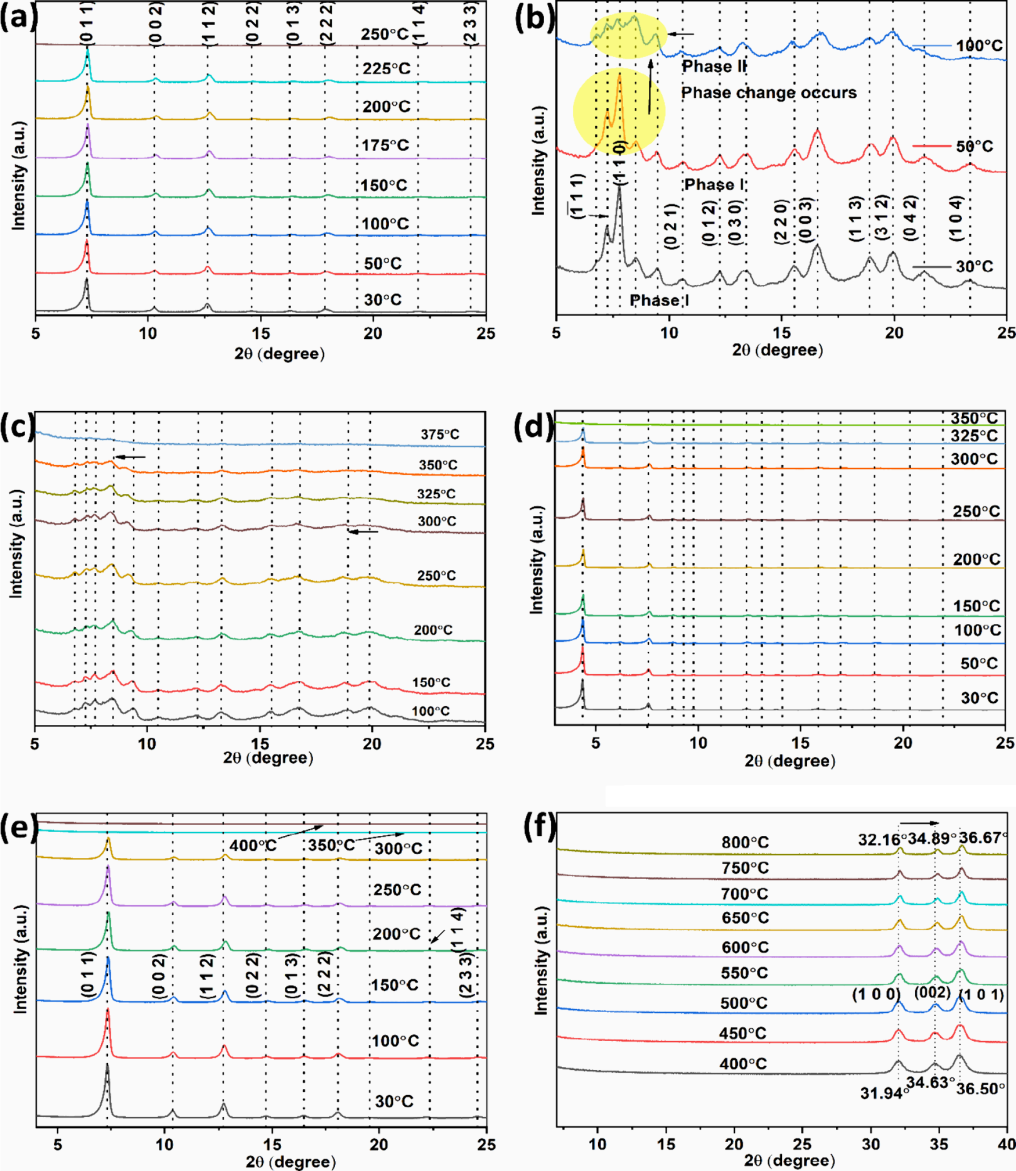
*In situ* heating XRD study of structural properties of (a) ZIF-67, (b,c) ZIF-7, (d) ZIF-71, and (e,f) ZIF-8 particles.

XRD patterns for ZIF-67 deposited on a Si substrate, measured at different temperatures in air, are illustrated in Figure 4a. The Miller indices (*h k l*) corresponding to specific planes were assigned in the 2θ range from 5 ° to 25 °. Similar assignments of Miller indices have been previously reported.^52^ A loss in XRD intensity is observed at 250 °C, indicating a loss of crystallinity and the onset of slow decomposition of the ZIF-67 framework due to thermal stress. With a further increase in temperature, the disappearance of ZIF-67 reflections and the emergence of Co_3_O_4_ reflections are observed at 250 °C and higher. A slight rightward shift in XRD reflections, within the range of 0.04–0.12 °, occurs as the temperature increases from 30 to 225 °C. This shift suggests a shrinking of the ZIF-67 framework. While similar observations have been reported previously; however, our results provide additional insight into the rightward peak shift induced by thermal stress, which deviates from standard behavior.^7^ This unusual thermal property of ZIF-67 is the occurrence of negative thermal expansion, which may be attributed to the soft vibrational or low-frequency transverse vibrational modes of the organic framework.^62^ This can cause the hinged framework to contract slightly instead of expanding during heating. At elevated temperatures ≥275 °C, the transformation of ZIF-67 into Co_3_O_4_ is evident, as shown in Figure S4­(a).

XRD patterns for ZIF-7 deposited on a Si substrate and tested at different temperatures in air are illustrated in Figure 4b,c. Diffraction reflections corresponding to phase-I of ZIF-7 at lower *in situ* temperatures (30 and 50 °C) were assigned as (−1 1 1), (1 1 0), (0 2 1), (0 1 2), (0 3 0), (2 2 0), (0 0 3), (1 1 3), (3 1 2), (0 4 2), and (1 0 4) planes at 7.23 °, 7.77 °, 10.58 °, 12.24 °, 13.4 °, 15.55 °, 16.57 °, 18.9 °, 19.92 °, 21.33 °, and 23.37 °, respectively, as also reported in other studies.^50^

By increasing the temperature from 50 to 100 °C, a phase change occurs, and ZIF-7 (phase-I) transforms into ZIF-7 (phase-II) with a few additional reflections at 6.77 ° and slight shift in some other reflections at lower 2θ, as shown in Figure 4b. This phase change is attributed to the release of synthesis or dispersion solvent molecules (i.e., DMF or methanol), driven by desorption from the framework, and subsequent structural rearrangement. Such rearrangements may correspond to modifications in the torsional strain and tilting of Zn–N coordination bonds, and the associated bond energy cost explains the gate-opening effect.^63^

A loss in diffraction intensity is observed at 325 °C (Figure 4c), confirming the loss of crystallinity and the slow decomposition of the ZIF-7 (phase-II) framework due to thermal stress. Above 50 °C, structural rearrangement occurs in the ZIF-7 framework, leading to a phase change and a loss of symmetry in the crystal structure. Beyond this temperature, an incremental loss of long-range order for Miller indices above 10 ° (2θ) is observed at 325 °C. With increasing temperature, an almost complete loss of ZIF-7 reflections was observed at 375 °C, followed by the emergence of ZnO reflections at 400 °C (Figure S4­(b)). Moreover, a slight leftward shift in additional ZIF-7 reflections is observed from 9.47 to 9.04 ° by increasing the temperature from 30 to 350 °C, which may be associated with the thermal expansion of the porous framework. The crystal structure retains its ZnO phase at 500 °C and does not revert even after cooling back to 30 °C, as illustrated in Figure S4­(b). Similar observations have been previously reported.^64^ However, our results provide additional information, particularly regarding the rightward shift in reflections due to thermal stress (Figure 4b), and reveal a loss of ZIF-7 reflections above 375 °C, which was not observed in previously reported literature.^64^ Similar studies have investigated the effect of temperature via TGA analysis, also confirmed by the previously reported study.^38^ Additionally, phase changes in ZIF-7 at higher temperatures have been observed in prior research.^50,63^

XRD patterns for ZIF-71 deposited on a Si substrate and measured at different temperatures in air are illustrated in the Figure 4d. A gradual loss in XRD intensity can be observed as the temperature increases from 30 to 325 °C, confirming the loss of crystallinity and the slow decomposition of the ZIF-71 framework due to thermal stress. With further increase in temperature, a near-complete loss of ZIF-71 reflections and the emergence of ZnO reflections are observed at 350 °C. Significant amorphization and reflection broadening are observed as the temperature increases from 30 to 325 °C. However, with the emergence of ZnO reflections above 325 °C, peak broadening decreases and crystallinity improves as more ZnO forms at higher temperatures (Figure S4­(c)). Our results provide additional insights, particularly regarding the peak shift to higher angles from 4.36 to 4.40 ° by increasing the temperature from 30 to 325 °C. This anomalous behavior, characterized by the negative thermal expansion, can be attributed to the soft lattice vibrations within the organic framework.^62^ After the transition to ZnO, the positive thermal expansion was observed by increasing the temperature from 375 to 500 °C.

Comparative XRD patterns for ZIF-8 deposited on a Si substrate, measured at different temperatures in air, are illustrated in the Figure 4e,f. A loss in XRD intensity is observed at 300 °C, confirming the loss of crystallinity and the slow decomposition of the ZIF-8 framework due to thermal stress. Beyond this temperature, an incremental loss of long-range order for Miller indices above 13 ° (2θ) is observed at 325 °C. With further increasing temperature, a near-complete loss of ZIF-8 reflections and the emergence of ZnO reflections are observed at 325 °C.

Notable amorphization and peak broadening were observed as the temperature increased from 30 to 325 °C. However, with the emergence of ZnO reflections above 325 °C, peak broadening decreases and crystallinity improves as more ZnO is formed at higher temperatures. A slight rightward shift in ZIF-8 reflections, by ∼0.12 °, is observed as the temperature increases from 30 to 325 °C (Figure 4e). This change at higher angles can be attributed to the release of guest molecules from the porous ZIF-8 framework, implying a shrinking structure. The most significant decrease in reflection intensity is observed for the (0 1 1) plane as the temperature increases from 30 to 300 °C, which is related to the Zn–N bond, and it leaves behind the 2-methylimidazolate linker. This observation is consistent with a previous report.^7^ Additionally, during the transformation of ZIF-8 into ZnO at higher temperatures (>325 °C), a further rightward shift of XRD reflections, by ∼ 0.20 °, is observed as the temperature increases from 400 to 800 °C, as shown in Figure 4f.

Similar observations have been reported in prior research.^65^ Our results provide additional information, particularly the rightward peak shift due to thermal stress, which is not standard behavior. Our findings also indicate a loss of ZIF-8 reflections above 325 °C, which is significantly higher than previously reported results.^65^ Similar studies have examined the effect of temperature on Raman shift or the effect of pressure on XRD reflections, as reported in literature.^4,41,66,67^

It is noted that the organic linker used is the same in both ZIF-8 and ZIF-67, with the difference lies in the central atom: Zn in ZIF-8 and Co in ZIF-67. For ZIF-8, thermal decomposition occurs at 325 °C, whereas in ZIF-67, thermolysis occurs much earlier at 250 °C. It has been reported that the metal–ligand bond in ZIF-8 is more easily broken under thermal heating compared to ZIF-67. This can be attributed to the higher bond energy of Co–N (2.834 eV) compared to Zn–N (2.075 eV), due to the higher electronegativity of Co relative to Zn.^68^

However, *in situ* XRD measurements under incremental temperature step-size variation-based isothermal XRD pattern showed that the ZIF-67 framework deteriorates at lower temperatures (250 °C) compared to ZIF-8 (325 °C). This observation cannot be fully explained by bond energy differences alone, indicating the need for deeper insights about the coordination chemistry of Zn and Co with nitrogen. The outer shell configuration of cobalt is 3d_7_4s_2_, indicating an incomplete shell, whereas Zn has a fully filled outermost shell (3d_10_4s_2_). This results in unsaturated coordination of Co with N, while Zn forms a saturated coordination with N. Consequently, Co–N bonds are more prone to oxygenation during heating compared to Zn–N, leading to faster thermolysis kinetics of ZIF-67 at lower temperatures. These results are further supported by TGA analysis.

A summary of the *in situ* XRD measurements for all four tested ZIFs is presented in Table 3, noting four temperatures (T1–T4) at which changes in XRD results have occurred:

1. T1 is the temperature up to which good intensity of ZIF reflections exists for phase-I.
2. T2 is the temperature at which another phase of ZIF emerged.
3. T3 is the temperature, where both ZIF and metal oxide phases coexist, i.e., the transition temperature.
4. T4 is the temperature at which complete degradation of ZIF framework and the emergence of metal oxide phase is clearly visible.

**Table 3:** Summary of Phase Changes and Framework Degradation

S. No.	material	temperature 1, T1 (°C)	temperature 2, T2 (°C)	temperature 3, T3 (°C)	temperature 4, T4 (°C)	comments
1.	ZIF-8	300		325	350	single phase of ZIF-8 exists and rightward peak shift was observed at higher temperatures, implying shrinking frame.
2.	ZIF-7	50	100	375	400	two phases of ZIF-7 exists and by increasing the temperature; the leftward shift at the smaller angle shows thermal expansion of the porous structure. On the contrary, at higher angles, the rightward shift shows thermal stress.
3.	ZIF-71	325		350	375	single phase of ZIF exists. Significant amorphization and peak broadening were observed by increasing a temperature, from 30 to 325 °C.
4.	ZIF-67	225		250	275	single phase of ZIF-67 exists. Slight shift in ZIF-67 peaks in rightward direction within the range of 0.04–0.12°, while going from the temperature of 30–225 °C. This small rightward shift, implying a shrinking frame of ZIF-67

### 3.3 Raman Analysis

The Raman spectra of the 0A sample set were measured in the range of 0–900 cm^–1^, as shown in Figure S5­(a). ZnO possesses a hexagonal wurtzite structure with no center of symmetry and belongs to the **C**_**6v**_^**4**^ space group.^69^ The optical phonon symmetric modes of the ZnO structure, as predicted by group theory, can be represented by the irreducible representation:^70^

$$\Gamma_{\mathrm{optical}}=A_{1}+2B_{1}+E_{1}+2E_{2}$$ 6

Among these four modes, *A*_1_ and *E*_1_ are polar modes, which can split into longitudinal optical (LO) and transverse optical (TO) components. The *A*_1_ mode corresponds to the Zn–O stretch along the *c*-axis. The *A*_1_ (TO) and *A*_1_ (LO) modes are observed at 381.56 and 582.9 cm^–1^, respectively. The lattice vibration symmetry is associated with the *A*_1_ (TO) mode, while the *A*_1_ (LO) mode provides information about electron–phonon interactions.^71^

Nonpolar modes observed in the Raman spectra include *E*_2_^low^ (98.28 cm^–1^), *E*_2_^high^ (438.62 cm^–1^), and the difference mode *E*_2_^high^–*E*_2_^low^ (331.81 cm^–1^), which vibrate perpendicular to the *c*-axis. The *E*_2_^low^ vibrational mode is associated with the heavier Zn sublattice at low frequencies (98.28 cm^–1^), while the *E*_2_^high^ mode corresponds to the lighter O sublattice at high frequencies (438.62 cm^–1^).

The Raman spectra of the M0 sample were measured over the same spectral range of 0–900 cm^–1^, as shown in Figure S5­(b). The Cd-doped ZnO sample exhibits characteristic modes similar to those of the 0A sample. However, due to Cd doping, both nonpolar and polar optical phonon modes exhibit a slight rightward shift. The *E*_2_^low^ (101 cm^–1^), *E*_2_^high^ (440 cm^–1^), and *A*_1_(LO) (584 cm^–1^) modes are observed in the Cd-doped ZnO Raman spectra. This can be attributed to increased lattice stress caused by the incorporation of larger Cd atoms into the ZnO lattice, as reported previously.^55^

The Raman scattering spectra of ZnO-based samples with four different ZIF coatings (ZIF-67, ZIF-7, ZIF-71, and ZIF-8)—1A, 2A, 3A, and 4A—along with their corresponding vibrational modes, are discussed in detail.

The vibrational modes present in the Raman spectra of the 1A film are shown in Figure 5a. A notable band near 339 cm^–1^ corresponds to the *E*_2_^high^–*E*_2_^low^ mode, which is a second-order acoustic mode.^72^ The vibrational band observed at 379 cm^–1^, corresponds to the *A*_1_(TO) phonon mode. The *A*_1_ vibrational mode is Raman active, and its vibrations polarize the unit cell. These vibrations generate an electrostatic field within the unit cell, causing the mode to split into longitudinal optical (LO) and transverse optical (TO) phonon modes.^73^ Another phonon mode observed at 611 cm^–1^, corresponds to the simultaneous interaction of transverse acoustic and transverse optical (TA + TO) phonon modes.^72^ This mode provides insight into second-order Raman scattering and offers information about the lattice dynamics of the crystal. The appearance of 2A1­(LO) band at 1140 cm^–1^ in the Raman spectra corresponds to the optical overtone mode of the ZnO lattice.^74^

**Figure 5:**
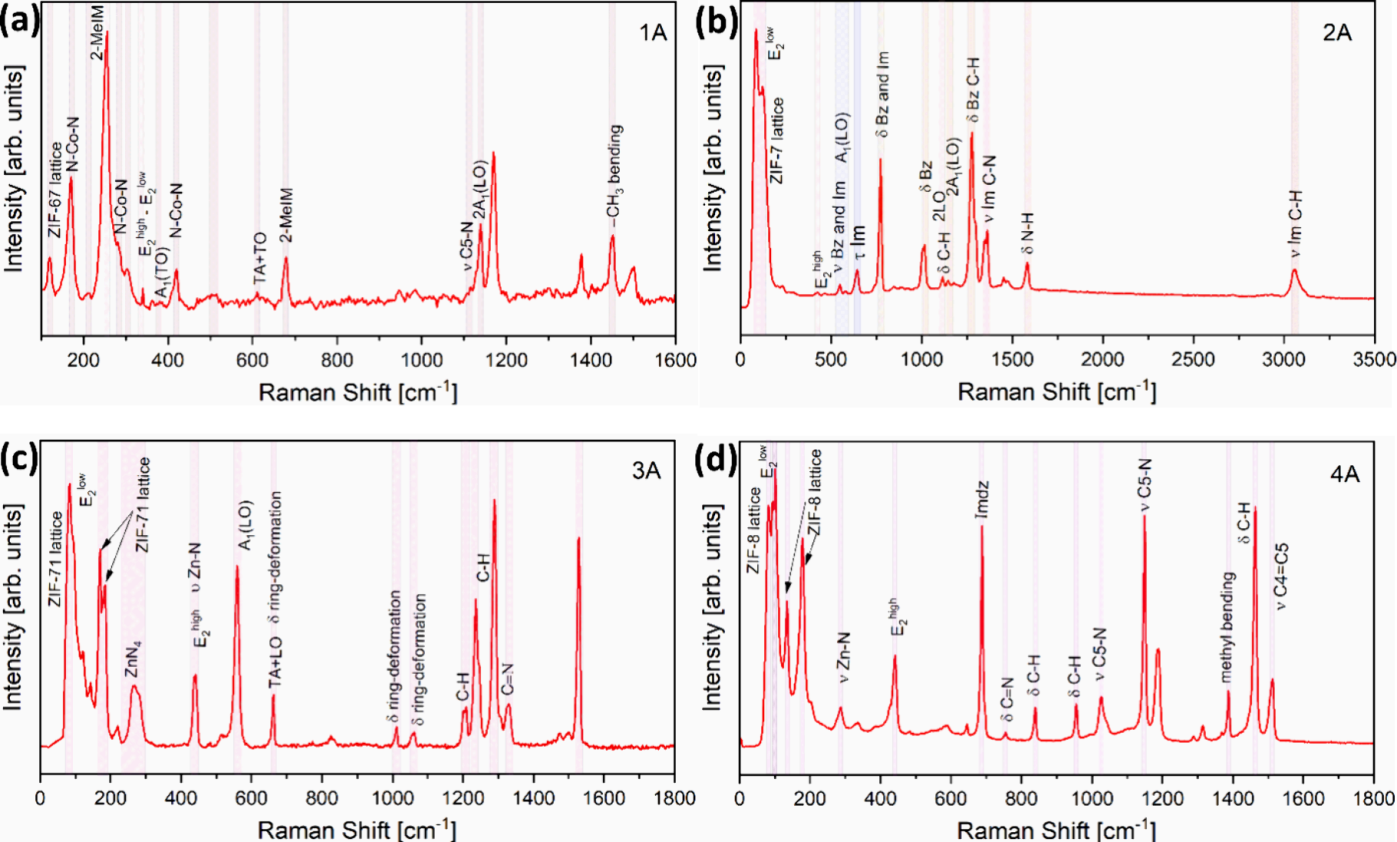
Raman spectra of the ZIF-coated ZnO samples: (a) 1A (ZIF-67-coated ZnO), (b) 2A (ZIF-7-coated ZnO), (c) 3A (ZIF-71-coated ZnO), and (d) 4A (ZIF-8-coated ZnO) sets.

Notable vibrational modes for ZIF-67 are present in the Raman spectra at 120, 171, 256, 281, 419, 679, 1110, and 1452 cm^–1^. These are associated with lattice vibrations, N–Co–N bending, linker vibrations, N–Co–N bending, N–Co–N bending, linker vibrations, υ-C_5_–N stretching, and methyl bending vibrations, respectively, as confirmed by previously reported studies.^75^

The significance of these bands can be explained in detail. The lattice vibration mode observed at 120 cm^–1^ include breathing modes, structural transitions, and instabilities, providing insights into lattice dynamics, in line with prior studies.^75^ Linker (2-methylimidazole) vibrations at 256 and 679 cm^–1^ confirm the successful synthesis of ZIF-67, supported by earlier studies.^76^ Metal–ligand (N–Co–N) vibrations were observed at 171, 281, and 419 cm^–1^, which are crucial for understanding the coordination between cobalt ions and ligands, corroborated by prior research.^76^ These peaks can indicate changes in the coordination environment. Other phonon modes at 1110 and 1452 cm^–1^ correspond to the stretching (υ) mode of C_5_–N in the imidazole ring and methyl bending vibrations in the imidazole ring, respectively, consistent with earlier investigations.^75^ These phonon modes can influence the adsorption capacity of the framework for guest molecules.

The Raman spectrum of the fabricated 2A sample is shown in Figure 5b, revealing characteristic vibrational modes associated with both ZIF-7 and ZnO structures. ZnO vibrational modes, observed at about 99, 429, 576, 1115, and 1156 cm^–1^ were assigned to *E*_2_^low^, *E*_2_^high^, *A*_1_(LO), 2LO, and 2*A*_1_(LO), respectively, consistent with previously reported study.^72^ The relatively low intensity of the ZnO modes is attributed to the presence of a ZIF-7 overlayer. Notably, some overlap occurs between ZnO and ZIF-7 modes, specifically *E*_2_^low^ with the ZIF-lattice, *A*_1_(LO) with the stretching mode of the linker, and 2LO with in-plane bending, as depicted in the Figure 5b.

The ZnO modes indicate its wurtzite crystal structure. The intense *E*_2_^low^ band is attributed to the vibrations of the Zn sublattice,^77^ while the lower-intensity *E*_2_^high^, *A*_1_(LO), 2LO, and 2*A*_1_(LO) bands correspond to vibrations of the O sublattice; structural faults due to O vacancies and second-order optical phonon modes involve longitudinal optical phonons, respectively.^77^ The *A*_1_ polar mode splitting, resulting in the LO phonon mode, and the 2LO process provide insights into phonon interactions and electron–phonon coupling within the ZnO crystal.^78^

ZIF-7 lattice framework modes were detected at 83.9 and 121.6 cm^–1^, consistent with a reported study.^79^ These modes are sensitive to phase transitions and structural changes within the ZIF-7 framework.^63^ Stretching vibrational modes (υ) at 547.3 and 1345 cm^–1^ were assigned to bond stretching within the benzimidazolate (Bz) and imidazolate (Im) rings, respectively.^79^ The torsion vibrational mode (τ), observed at 644.3 cm^–1^, corresponds to twisting and rotational movements of the Im ligand and involves changes in the angles between the ligand and the metal ring.^63^

The Raman spectrum of the fabricated 3A sample set is shown in Figure 5c. The spectrum exhibits several characteristic vibrational modes corresponding to the ZIF-71 and ZnO structures. ZnO vibrational modes observed at 96, 439, 560, and 662 cm^–1^ are assigned to *E*_2_^low^, *E*_2_^high^, *A*_1_(LO)*,* and TA + LO, respectively, in accordance with previously published data.^77^ Two moderately intense nonpolar phonon modes (*E*_2_^low^ and *E*_2_^high^) are attributed to vibrations of the heavy Zn sublattice and the lighter oxygen sublattice, respectively, in the ZnO crystal.^78^ Other phonon modes, such as *A*_1_(LO) and TA + LO, are attributed to structural faults due to oxygen vacancies^77^ and simultaneous interactions of transverse acoustic and longitudinal optical phonon modes,^72^ respectively.

Vibrational modes corresponding to the ZIF-71 lattice framework structure modes were observed at 84, 169.7, and 183.9 cm^–1^, assigned to the ZIF-71 lattice framework, as confirmed by previously reported studies.^80^ These modes are sensitive to structural changes and phase transitions, providing insight into lattice dynamics. Modes associated with the tetrahedra configuration of metal–ligand interactions (ZnN_4_) and Zn–N bond stretching vibrations were observed at approximately 266.6 and 422.3 cm^–1^, respectively, in line with prior studies.^80^ Additional modes corresponding to the dicholoroimidazolate ring were observed at 660.9, 1011.3, and 1054.9 cm^–1^, associated with in-plane (δ) linker ring deformation.^81^ Higher wavenumber phonon modes at 1209.4, 1236, 1288, and 1328 cm^–1^ correspond to C–H and C=N bending (δ) and stretching (υ) vibrations, respectively.^80,82,83^

The Raman spectrum of the fabricated 4A sample set is shown in Figure 5d. The spectrum exhibits several characteristic vibrational modes corresponding to the ZIF-8 and ZnO structures. ZnO vibrational modes at 102 and 442 cm^–1^ are assigned to *E*_2_^low^ and *E*_2_^high^, respectively, in agreement with previous reports.^77^ Two moderately intense nonpolar phonon modes (*E*_2_^low^ and *E*_2_^high^) correspond to the vibrations of heavy Zn sublattice and the lighter oxygen sublattice in the ZnO crystal.^78^

ZIF-8 lattice framework vibrational modes were observed at 82, 135, and 179 cm^–1^, consistent with the previous report.^75^ These modes are sensitive to phase transitions and structural changes in the ZIF-8 framework, providing insights into lattice dynamics.^75^ Stretching vibrational modes (υ) were observed at 285, 1025, 1146, and 1512 cm^–1^, assigned to stretching (υ) of bonds between the metal ligand and imidazole ring (Zn–N), C_5_–N in the imidazole ring, C_5_–N in the imidazole ring, and C4 = C5 in the imidazole ring, respectively.^84^ A moderately intense methyl bending band at 1388 cm^–1^ is attributed to the imidazolate ring in the ZIF-8 framework.^85^ A strong band at 685 cm^–1^ corresponds to the out-of-plane bending of the imidazolate ring. Vibrational bands corresponding to in-plane bending (δ) were observed at 754, 839, 957, and 1463 cm^–1^, assigned to C=N, C–H, C–H, and C–H in the imidazolate ring, respectively.^85^

Similarly, Raman analysis was performed on the M1, M2, M3, and M4 samples, exhibiting Raman bands similar to those of the corresponding undoped counterparts, as shown in Figure S6. However, a slight shift in bands was observed for the Cd-doped ZnO samples.

For instance, the vibrational modes of sample M1 are observed at lower frequencies compared to those of sample 1A. Figure S7­(a) presents a comparison of the vibrational spectra of sample M1 and sample 1A within the spectral range of 0–1600 cm^–1^. Figure S7­(b) shows a close-up view, clearly distinguishing two peaks. The 2*A*_1_(LO) vibrational peak was observed at 1139.87 cm^–1^ for sample 1A, shifting by 1.68 to 1141.55 cm^–1^ for sample M1. This shift can be attributed to increased lattice stress caused by the incorporation of large Cd ions into the ZnO lattice, as previously reported.^55^ The 2*A*_1_(LO) mode was chosen because it provides insight into doping effects through electron–phonon interactions.

### 3.4 XPS Analysis

To analyze the surface chemistry of the different ZIF-coated Cd-doped ZnO film MOF/MO_*x*_ samples, XPS was performed on a set of M1–M4 samples. Figure 6 shows the corresponding survey scans with labels indicating the photoelectron and Auger electron peaks. Each spectrum shows C 1s, N 1s, O 1s, and Zn 2p lines, indicating that each sample surface contains the elements C, N, O, and Zn. Moreover, the M1 sample spectrum corresponding to a ZIF-67 coating exhibits a pronounced Co 2p signal, which implies the presence of Co. Additionally, the M3 sample spectrum contains distinct Cl 2s and Cl 2p signals, indicating the presence of Cl at this sample surface. A summary of the surface compositional analysis is given in Table S1. The presence of Zn in the M2–M4 samples is expected, since, here, the corresponding ZIF structures contain Zn as the metal part of the framework.^86–88^ Furthermore, the Cl content in the M3 sample most likely associates with the dichloroimidazolate organic linker present in the corresponding ZIF-71 framework.^89^ The relatively high amount of Co in the M1 sample also matches with the expectation because in the corresponding ZIF-67 framework, a Co ion is the coordinated metal ion.^90^ However, the presence of Zn in the M1 sample strongly indicates that the coating does not cover the entire ZnO substrate surface. This could be due to the drop-casting method, which might lead to inhomogeneous thickness and potential voids. Another substrate influence is observed in the M3 sample associated with ZIF-71. Here, the Zn 2p signal possesses distinct side peaks that we associate with the ZnO substrate at a different surface charge. A Cd signal is not observed in each survey spectrum, which is most likely due to the low-doping concentration in the underlying ZnO substrate.

**Figure 6:**
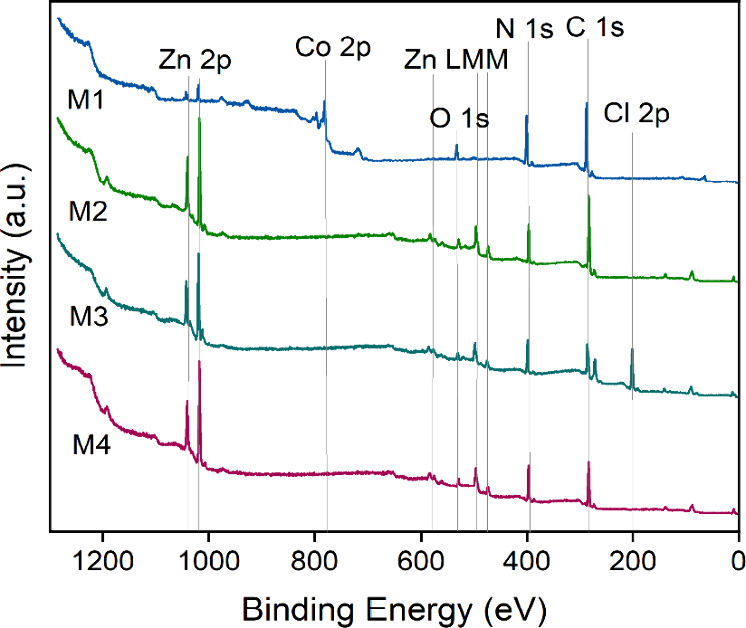
XPS survey spectra of ZIF-coated Cd-doped ZnO samples: M1 (ZIF-67), M2 (ZIF-7), M3 (ZIF-71), and M4 (ZIF-8).

To further analyze and validate the surface chemistry of the ZIF coatings, XPS high-resolution scans were performed on each sample. Figure 7 shows the fitted metal 2p as well as the fitted N 1s and C 1s spectra of each sample. For the M3 sample containing Cl, Figure 7M3d adds the corresponding Cl 2p spectrum. Despite applying the charge correction as detailed in the experimental section, the N 1s, O 1s, and C 1s photoemission peaks of each sample as well as the Cl 2p peak of the ZIF-71 containing the M3 sample show shifts toward higher binding energies (*E_b_* s) compared to previously reported *E_b_* s for similar ZIF-coated materials.^1,47^ As an example, Figure S8 shows a comparison between the charge-corrected high-resolution scans of the M3 sample from this study with similarly charge-corrected high-resolution scans of a ZIF-71-coated CuO:Al film from a previous study.^47^ The *E_b_* shifts of Δ*E_b_* of the main components are all toward higher *E* *_b_* s and range from Δ*E*_*b*_≈ +1.9 eV for the C 1s peak to Δ*E*_*b*_≈ +1.1 eV for the O 1s peak. We attribute these shifts to differential charging effects, i.e., inhomogeneous charging of the sample surface and are currently further investigating this effect and its origin. Despite these shifts, we assume that the spectra can still be subjected to a consistent analysis because the peak shapes allow a direct comparison. Therefore, the following analysis focuses on the position of fitting components within a peak rather than absolute *E_b_* positions. Despite this, the absolute *E_b_* positions of all fitting functions presented in the analysis below are documented in Tables S2M1–M4.

**Figure 7:**
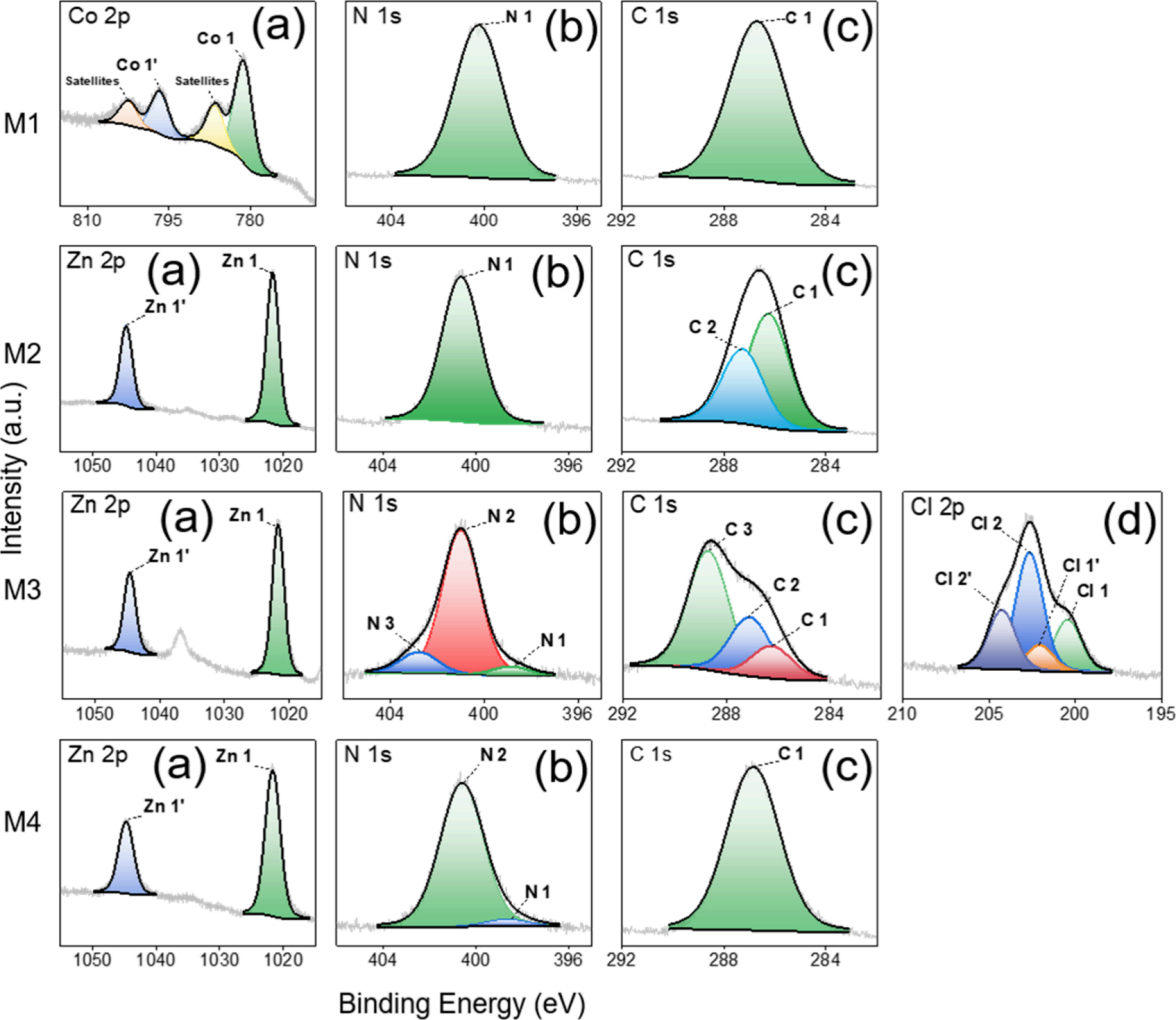
XPS high-resolution spectra of ZIF-coated Cd-doped ZnO samples: M1a–M1c: ZIF-67, M2a–M2c: ZIF-7, M3a–M3d: ZIF-71, and M4a–M4c: ZIF-8.

Figure 7M1a–M1c shows the most relevant high-resolution spectra of the M1 sample associated with ZIF-67. The Co 2p spectrum in Figure 7M1a exhibits a characteristic spin–orbit splitting into a Co 2p_1/2_ component at higher *E_b_* (Co 1′) and a Co 2p_3/2_ component at lower *E_b_* (Co 1). Both components show a main peak with the addition of a subpeak at higher *E_b_* s. While the main peaks most likely associate with the Co^2+^ ion in the ZIF-67 structure, the subpeaks at higher *E_b_* s are satellites related to shakeup processes.^49^ The N 1s spectrum shown in Figure 7M1b is fitted with a single component named N 1. This indicates that the N at the M1 sample surface is present in one dominant bonding environment. Here, it is highly likely that this single component associates with the N present in the ZIF-67 imidazolate linkers, which bond to Co at the N-sites. The C 1s spectrum displayed in Figure 7M1c also shows only one component that most likely associates with the C–N/C=N bonds of the imidazolate. In summary, the high-resolution Co 2p, N 1s, and C 1s spectra of the M1 sample indicate the presence of an intact ZIF-67 layer at its surface.

Figure 7M2a–M2c displays the high-resolution spectra of the M2 sample, which associates with a ZIF-7 coating. Here, the Zn 2p spectrum shown in Figure 7M2a exhibits a characteristic spin–orbit splitting into a Zn 2p_3/2_ lower *E_b_* component (Zn 1) and a Zn 2p_1/2_ higher *E_b_* component (Zn 1′). At first glance, a clear distinction between the Zn originating from the ZIF-7 and the Zn originating from the ZnO substrate is not possible since the oxidation states of the Zn ion are the same at Zn^2+^ in both bonding environments.^91^ However, as already indicated by the survey scan for the M3 sample in Figure 6 and later discussed for the M3 sample Zn 2p spectrum with respect to Figure 7M3a, the ZnO substrate is highly likely to be subjected to a different charging state. This leads to additional subpeaks with severe *E_b_* shifts in the Zn 2p signal when a substrate influence is present. Since additional Zn 2p photoelectron peaks are not observed in the Zn 2p spectrum of the M2 sample, the substrate influence is most likely negligible in this case. However, to further validate the presence of ZIF-7 at the sample surface, the N 1s and C 1s spectra must be considered as well. Similarly to the M1 sample, the N 1s peak of the M2 sample in Figure 7M2b exhibits only one component (N 1), which likely associates with the C–N/C=N bonds of the imidazolate. The C 1s peak from Figure 7M2c is fitted with a high *E_b_* component at lower intensity (C 2) and a low *E_b_* component at significantly higher intensity (C 1). While the high *E_b_* C 2 most likely associates with the C–N/C=N bonds of ZIF-7, we assign the low *E_b_* C 1 to the C–C/C=C bonds. These bonds are part of the additional aromatic ring in the benzimidazole linkers present in ZIF-7 ^86^ and are therefore expected to outweigh the number of C–N/C=N bonds. This expectation matches with the higher intensity of the C–C/C=C associated low *E_b_* component. Consequently, the C 1s and N 1s spectra indicate an intact ZIF-7 layer at the M2 sample surface.

The most relevant photoelectron spectra of the M3 sample are shown in Figure 7M3a–M3d. As already pointed out above, the Zn 2p spectrum in Figure 7M3a shows an additional subpeak at *E_b_* s in between the expected spin–orbit split Zn 2p_1/2_ (Zn 1′) and Zn 2p_3/2_ (Zn 1) components. Here, we associate this subpeak with the Zn 2p_1/2_ signal from the ZnO substrate. It is likely that the ZnO substrate possesses a different charging state than the ZIF-71 layer, and variance in thickness or pinholes in the ZIF-71 layer caused a strong differential charging effect on the sample, which is now observed by the *E_b_* shifted substrate influence in the Zn 2p spectrum. Similar to the ZIF-71 layer from a previously reported study, the N 1s spectrum from Figure 7M3b exhibits three components named N 1, N 2, and N 3.^47^ While the N 2 component at intermediate *E_b_* exhibits the highest intensity, the N 1 and N 3 components show noticeably lower intensities. The highest *E_b_* component N 3 relates to the Zn–N bonds in the ZIF-71 structure. This component has been reported to degrade under EUV light and X-rays.^47,92,93^ The N 2 components relate to N–H bonds while the N 1 component corresponds to C–N bonds.^92^ Both of these bonds might originate from fragmented imidazolate linkers, which are products of the X-ray degradation of ZIF-71.^92^ To further investigate this possible degradation, both the Cl 2p and the C 1s spectrum must be considered. The C 1s spectrum in Figure 7M3c shows three components named C 1, C 2, and C 3. The highest *E_b_* component C 3 most likely corresponds to intact imidazolate linkers in the nondegraded ZIF-71 while the lowest *E_b_* component C 1 probably associates with carbon-containing species present on the ZnO substrate subjected to a different surface charge. We associate the intermediate C 2 component to carbon that is not bonded to the electron-withdrawing partners. Here, the ZIF-71 degradation might have introduced such carbon-containing components. Furthermore, the Cl 2p spectrum from Figure 7M3d supports the interpretation of ZIF-71 degradation. The spectrum shows two doublets named Cl 2′, Cl 2 and Cl 1′, Cl 1. Both doublets associate with the spin–orbit splitting into Cl 2p_3/2_ at higher *E_b_* and Cl 2p_1/2_ at lower *E_b_* and match the overall arrangement of Cl 2p subpeaks reported in previous studies on ZIF-71 degradation.^47,92,93^ However, the higher *E_b_* Cl 2′, Cl 2 doublet corresponds to C–Cl bonds, which is present in intact ZIF-71, and the lower *E_b_* Cl 1′, Cl 1 doublet corresponds to Zn–Cl bonds.^47,92,93^ These bonds can originate from linker fragments that formed by ZIF-71 degradation reactions initiated by Cl radicals as reported.^92^ In summary, the high-resolution spectra of the M3 sample strongly indicate a significant degree of ZIF-71 degradation as well as a substrate influence on the surface.

Figure 7M4a–M4c shows the Zn 2p, C 1s, and N 1s spectra of the M4 sample, which correspond to a ZIF-8 coating. The Zn 2p spectrum from Figure 7M4a exhibits the characteristic spin–orbit split Zn 2p_1/2_ (Zn 1′) and Zn 2p_3/2_ (Zn 1) peaks. As explained already for M2 above, the substrate influence is most likely negligible in this case since no additional Zn 2p peak is observed. The C 1s spectrum shown in Figure 7M4b is fitted by a single component (C 1) that we associate with C–N/C=N bonds present in the ZIF-8 imidazolate linker. Additionally, the N 1s spectrum from Figure 7M4c shows a dominant N 2 component at high *E_b_* and a relatively low-intensity N 1 component at lower *E_b_*. While the N 2 component most likely corresponds to N bond to Zn^2+^ in the ZIF-8 structure, the N 1 component is related to uncoordinated 2-methylimidazole linkers.^1,94^

In addition to the elements presented in Figure 7, each sample contains a relatively small amount of oxygen. Figure S9 presents the corresponding high-resolution spectra of the M1–M4 samples. We associate the presence of oxygen with a small degree of surface oxidation and the adsorption of oxygen-containing carbon components since the samples were exposed to air during sample storage and fabrication. However, as seen from the compositional analysis presented in Table S1, the surface oxygen content does not exceed approximately 4.0 at%. Therefore, we assume the influence of oxygen on the ZIF surfaces to be rather small for the interpretation of the sensing properties presented in later sections.

### 3.5 Thermal Stability of ZIF-67, ZIF-7, ZIF-71, and ZIF-8 Particles

The thermal stability of bulk ZIF particles was evaluated by TGA measurements. Figure 8 shows the TGA curves of the four different ZIF samples. According to the TGA results, ZIF-7, ZIF-8, ZIF-71, and ZIF-67 are thermally stable up to approximately 480, 370, 410, and 330 °C, respectively. The final mass residues at 800 °C for ZIF-7, ZIF-8, and ZIF-67 were 34.1, 25.6, and 34.5%, respectively, which are consistent with their theoretical values of 35.4, 25.4, and 36.3%, corresponding to the formation of respective metal oxides, i.e., ZnO or Co_3_O_4_. In contrast, the final mass of ZIF-71 is 5.2%, significantly lower than the theoretical value of 23.4%. However, this result appears to align with the previously reported findings.^95,96^ The difference between the theoretical and experimental value could be attributed to the presence of extra 4,5-dichloroimidazole linkers coordinated to zinc atoms at the crystal surface.^97^ The thermal stability limits of ZIF-n (*n* = 67, 7, 71, and 8) were evaluated by correlating TGA data with the temperature-dependent *in situ* XRD patterns. The observed deviations in the upper stability thresholds between the two techniques are likely due to the higher ramping rates in TGA, which contrast with the prolonged thermal exposure times required for *in situ* XRD measurements at equivalent temperatures. Consequently, these stability ranges define the permissible operational temperature range for gas-sensing applications, ensuring the structural integrity of the ZIF-n frameworks during device operation.

**Figure 8:**
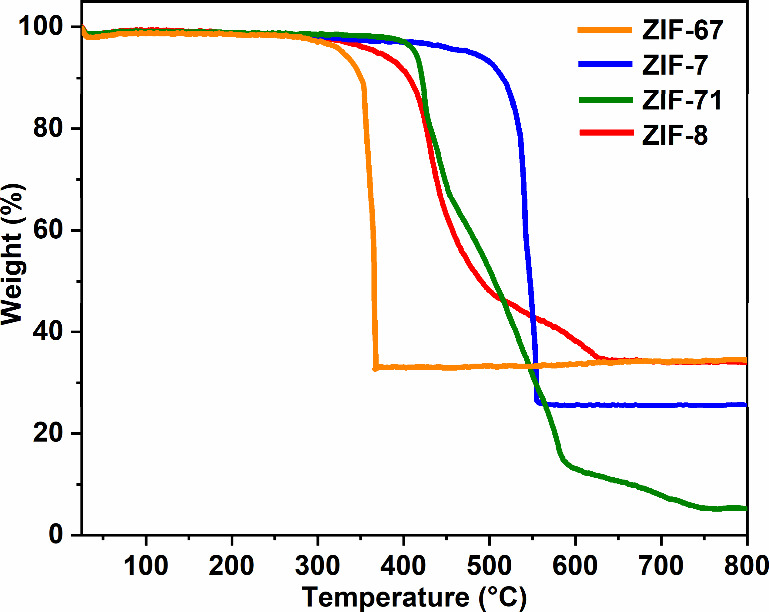
TGA analysis for thermal stability test of different ZIFs: ZIF-67, ZIF-7, ZIF-71, and ZIF-8.

### 3.6 Current–Voltage (I–V) Characteristics of MOF/MO_*x*_, Arrhenius Plots, and Trap Energy States

The I–V characteristics of the reference samples 0A and M0 are shown in Figure S10­(a,b), respectively. It shows a deviation from linearity at higher temperatures (300 °C) for the sample 0A. This transition is attributed to the thermal release of trapped carriers from defect states, such as interstitial zinc (Zn_i_) and zinc vacancies (*V*_Zn_), at elevated temperatures (300 °C). A similar mechanism of carrier release and abrupt increase in charge carrier density in the ZnO has been previously reported.^98^ Figure 9 plots the measured I–V characteristics of all the four samples: 1A, 2A, 3A, and 4A. At moderate temperatures, ranging from room temperature (RT, 20 °C) to 250 °C, the electrical contact between the Au IDEs and ZIF-coated ZnO films exhibits Ohmic behavior. Upon an increase in the temperature to 300 °C, a deviation from linearity is observed in the current–voltage characteristics for the Au contacts on the ZIF-coated ZnO films. This transition is attributed to the thermal release of trapped carriers from defect states, as discussed above in the case of reference samples.

**Figure 9:**
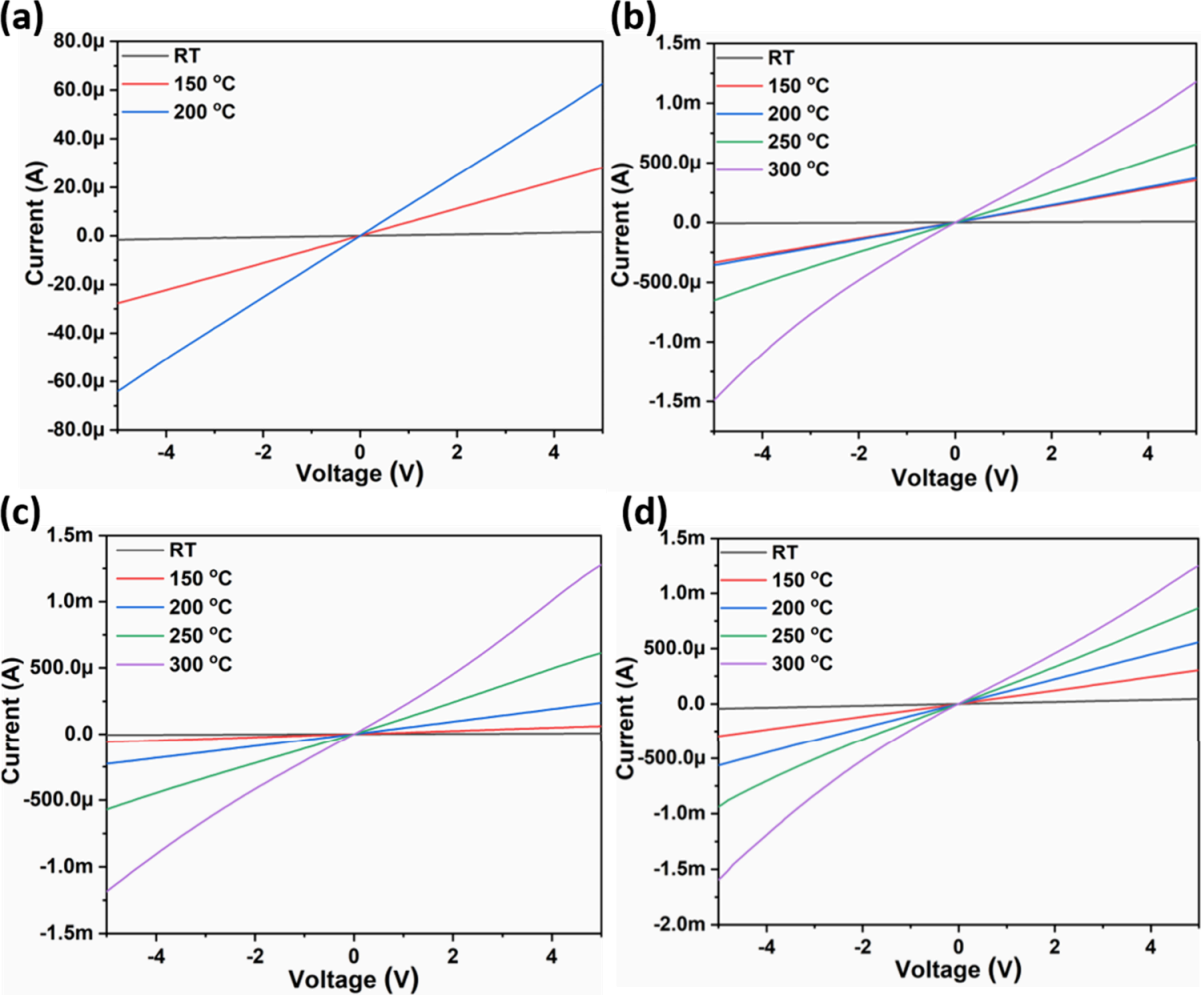
I–V characteristics of MOF/MO_*x*_: (a) 1A (ZIF-67-coated ZnO), (b) 2A (ZIF-7-coated ZnO), (c) 3A (ZIF-71-coated ZnO), and (d) 4A (ZIF-8-coated ZnO) samples at different temperatures.

Similarly, the I–V characteristics for a batch of Cd-doped ZnO samples (M1, M2, M3, and M4) are shown in Figure S11­(a–d). In these measurements, both forward and reverse voltage sweeps were employed to test for hysteresis behavior. An inverted hysteresis behavior was observed, which can be attributed to capacitive hysteresis; charge traps slow down the return path.^99,100^ This effect is strongly dependent on the voltage sweep rate.

For the ZIF-coated ZnO samples (labeled 1A to 4A), the Arrhenius plot derived from the temperature-dependent I–V characteristics is shown in Figure 10a. Similarly, the Arrhenius plot for Cd-doped ZnO samples (labeled as M1 to M4) is presented in Figure 10b.

**Figure 10:**
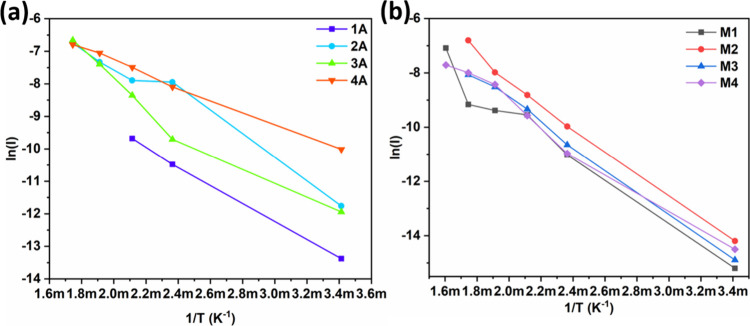
Arrhenius plot of two different batches of hybrid samples, (a) 1A (ZIF-67-coated ZnO), 2A (ZIF-7-coated ZnO), 3A (ZIF-71-coated ZnO), and 4A (ZIF-8-coated ZnO) and (b) M1 (ZIF-67-coated ZnO:Cd), M2 (ZIF-7-coated ZnO:Cd), M3 (ZIF-71-coated ZnO:Cd), and M4 (ZIF-8-coated ZnO:Cd) samples.

The Arrhenius equation relates electrical current to temperature, with activation energy (*E* *_a_*) calculated using:

$$I\left(T\right)=I_{o}e^{-E_{a}/k_{B}T}$$ 7

where *I*(*T*) is the measured current at the operating temperatures (T), *I* *_o_* is the pre-exponent factor, *E* *_a_* is the activation energy, and *k* *_B_* is the Boltzmann’s constant (8.617 eV/K).

Eq 7 can be linearized as

$$\ln\left(I\left(T\right)\right)=\left(\frac{-E_{a}}{k_{B}}\times \frac{1}{T}\right)+\ln\;I_{o}$$ 8

where *E* *_a_* */k* *_B_* is the slope of the straight line and *I* *_o_* is the intercept of ordinate axis.

Using linear regression on the data in Figure 10a,b, the slopes were extracted, and activation energies were calculated for all eight samples. The results are summarized in Table 4.

**Table 4:** Values of Slope of the Curve and Activation Energy for All the Eight Samples

sample name	slope of the curve (−*E_a_*/*k_B_*) in *K*	calculated activation energy (*E* *_a_*) in *eV*
1A	–2870.842	0.247
2A	–2964.291	0.255
3A	–3094.449	0.267
4A	–1950.181	0.168
M1	–4101.909	0.353
M2	–4301.349	0.371
M3	–4172.621	0.359
M4	–3895.565	0.336

The calculated activation energies are consistent with previously reported scientific values,^101^ corresponding to distinct defect levels. In all ZnO films, the presence of different ZIFs slightly alters the activation energies. Figure 11a illustrates the presence of all defect levels across the four samples. Among these, three samples (1A, 2A, and 4A) exhibit interstitial zinc (*Zn* *_i_*) level defects, with only slight variations in activation energies. On the other hand, sample 3A shows a trap carrier level corresponding to Zinc vacancies (*V* *_Zn_*).

**Figure 11:**
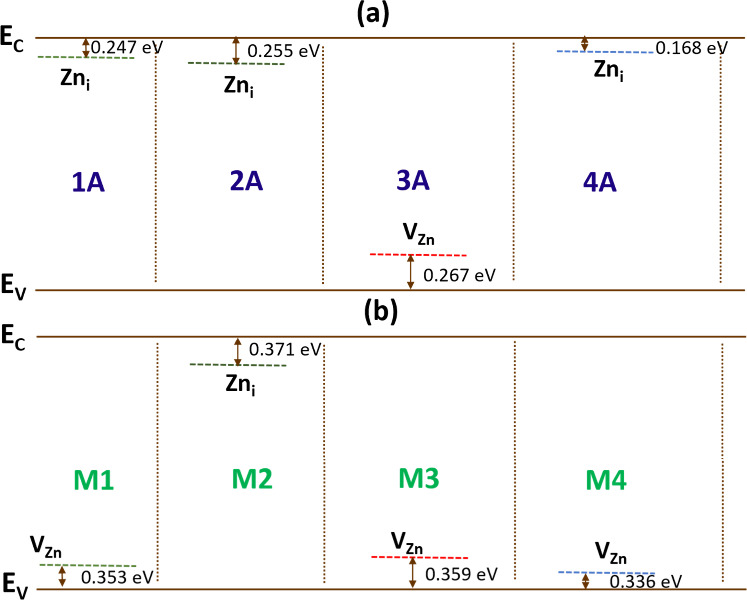
Trap energy states for carriers in different samples: (a) 1A (ZIF-67-coated ZnO), 2A (ZIF-7-coated ZnO), 3A (ZIF-71-coated ZnO), and 4A (ZIF-8-coated ZnO) and (b) M1 (ZIF-67-coated ZnO:Cd), M2 (ZIF-7-coated ZnO:Cd), M3 (ZIF-71-coated ZnO:Cd), and M4 (ZIF-8-coated ZnO:Cd).

For the ZIF-coated Cd-doped samples (M1–M4), three of them (M1, M3, and M4) exhibit intrinsic zinc vacancies (*V* *_Zn_*), while M2 displays interstitial Zn (*Zn* *_i_*) level defects, as shown in Figure 11b. The calculated activation energies—M1 (0.353 eV),^102^ M2 (0.371 eV),^103^ M3 (0.359 eV),^102^ and M4­(0.336 eV)^104^—are in good agreement with the literature values.

### 3.7 Gas-Sensing Properties of MOF/MO_x_ Hybrid Structures

The gas-sensing responses of the 0A and M0 samples to all tested gases were measured at various temperatures ranging from 20 to 300 °C, as shown in Figures S11­(a,b). For the 0A sample, the maximum gas-sensing responses were observed for n-butanol and 2-propanol at low temperatures (200 and 250 °C) and for ethanol at higher temperature (300 °C). These results are supported by the previously reported studies.^105–107^

Selective detection of homologous alcohol series is generally challenging; however, the present work demonstrates a possible approach using temperature modulation. For instance, Meng et al. reported selective detection of homologous alcohols using three different ZnO-based gas sensors by modulating the operating temperature, which was attributed to the regulation of oxygen vacancies at different temperatures.^108^

After Cd doping, gas response to hydrogen is known to improve, as reported in previous studies.^109^ For the M0 sample, at a low operating temperature (150 °C), the highest gas-sensing response was observed for n-butanol (∼3.7%) compared to other tested analytes. These findings align with prior reports indicating that ZnO particles exhibit selective sensitivity to n-butanol over ethanol, 2-propanol, and acetone.^110^

At higher operating temperatures (300 °C), the maximum sensing response was observed for hydrogen gas (∼15%). Enhanced hydrogen gas sensitivity at elevated temperatures has also been reported in earlier studies.^111–113^ A significant increase in sensitivity to n-butanol at low temperature (150 °C), along with the marked enhancement in hydrogen sensitivity at temperatures of ≥150 °C, can be attributed to the effect of Cd doping. These observations are further supported by earlier reported studies.^109,114^

**Figure 12:**
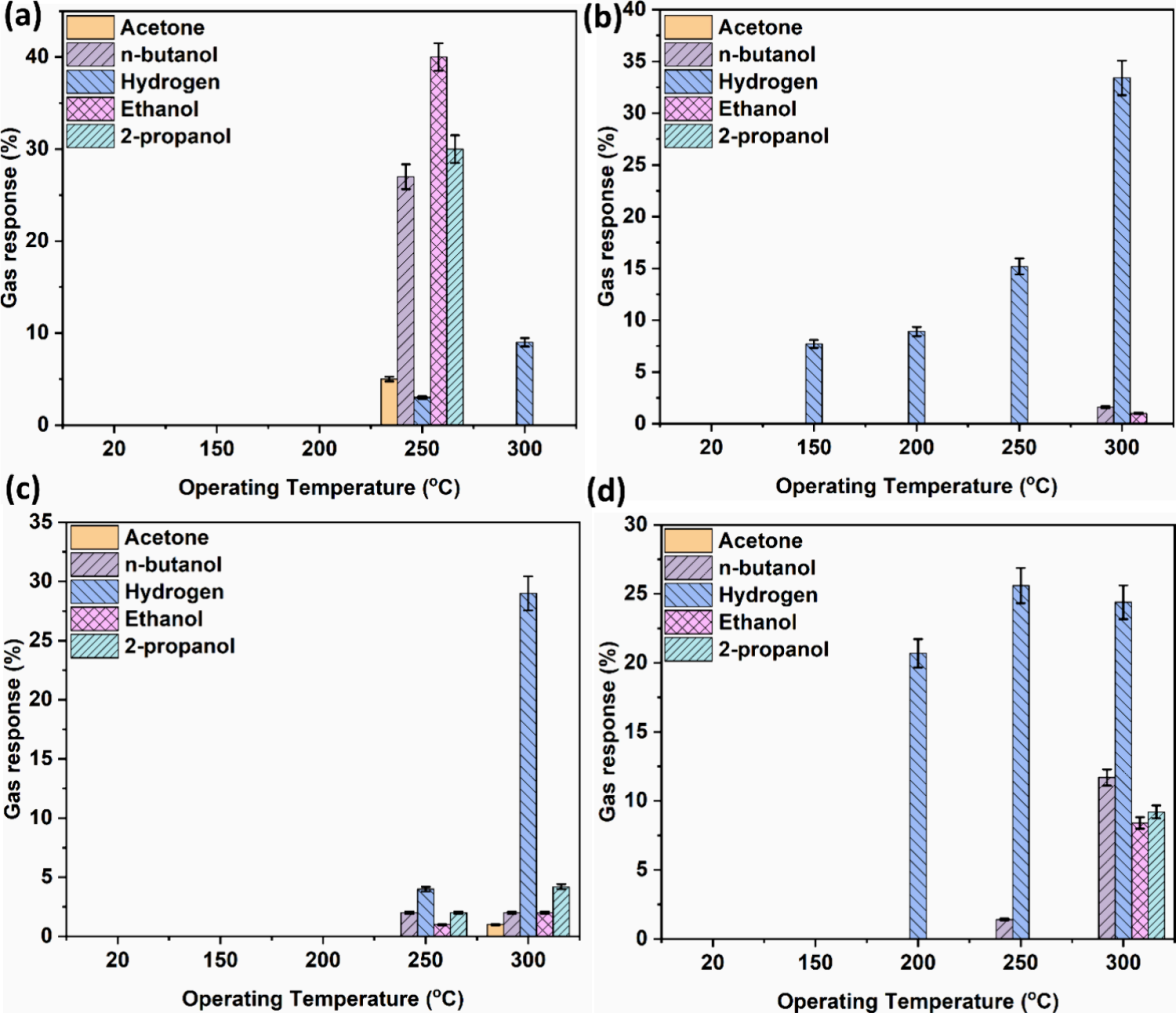
Gas-sensing results of different gases for MOF/MO_*x*_ hybrid structures: (a) 1A (ZIF-67-coated ZnO), (b) 2A (ZIF-7-coated ZnO), (c) 3A (ZIF-71-coated ZnO), and (d) 4A (ZIF-8-coated ZnO) samples, at different operating temperatures in the range of 20 to 300 °C.

In MOFs, target molecules are generally adsorbed through the pore structures or active sites. However, due to the absence of functional groups in ZIF-67, the only available adsorption pathway for target molecules is through its pore structure. The ZIF-67 framework offers two channels for molecular diffusion through its window apertures: a four-membered-ring (4R) aperture and a six-membered-ring (6R) aperture. The pore window size is 0.8 Å for the 4R aperture and 3.4 Å for the 6R aperture, as shown in Figure 13.

**Figure 13:**
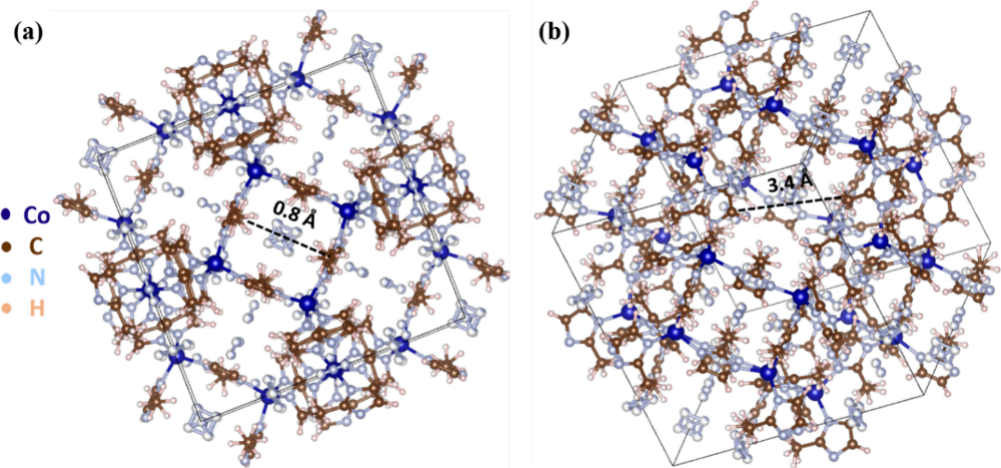
Pore window size of the ZIF-67 framework through two different membered rings: (a) a four-membered (4R) ring and (b) a six-membered (6R) ring. Crystal structure of ZIF-67 visualized using VESTA.^115^ Adapted with permission from ref 116 Copyright 2008 Royal Society of Chemistry.

The passage of target molecules through the window apertures of MOFs involves interaction between them that can be analyzed using Derouane’s theory.^117^ According to this theory, the effective van der Waals physisorption energy can be described by eq 9:^117^

$$F\left(f\right)=-\left(\frac{k}{4r^{3}}\right){\left(1-\frac{1}{2f}\right)}^{-3}$$ 9

where *F­(f)* is the van der Waals physisorption energy between a framework pore and target molecule; *k* is a molecular constant, which is directly related to the polarizability of the adsorbate; *r* is an effective interaction distance or adsorption distance between the pore wall and the center of molecule; *f (r/s)* is the curvature factor; and *s* is the pore window size.

This model results in three cases depending on the size of the target molecule.

First, when the size of the target molecule (*r*) is much smaller than the pore window size (*s*), an effective van der Waals interaction approaches zero. Second, when the molecular size is approximately equal to the pore window size, the van der Waals force is maximized, reaching up to eight times that of the first case (when *s* = 0). For the third case, if the molecular size exceeds the pore window size, a significant energy barrier arises, hindering the passage of molecule through the pores. Therefore, optimal adsorption occurs when the molecular size closely matches the pore window size.

The kinetic diameters of the tested molecules are acetone (4.6 Å),^118^ n-butanol (5.0 Å),^119^ hydrogen (2.89 Å),^27^ ethanol (4.53 Å),^119^ and 2-propanol (4.7 Å).^120^ For the 4R aperture of ZIF-67, the pore aperture size is 0.8 Å, which is too small for the passage of any of the tested analytes. Similarly, for the 6R aperture (3.4 Å), all tested analyte molecules are too large to pass through except for hydrogen. In temperature-dependent *in situ* XRD results, the transition from ZIF-67 to Co_3_O_4_ begins at 250 °C, where both phases coexist. For temperatures below 250 °C, the sensing mechanism of ZIF-67 dominates, resulting in the blockage of larger molecules and allowing only smaller molecules, such as hydrogen, to pass through. However, at temperatures of 250 °C or higher, the transformation of ZIF-67 to Co_3_O_4_ leads to the formation of a Co_3_O_4_/ZnO p-n heterostructure. Compared to the ZnO reference sample (0A), a significant increase in the sensing response for ethanol was observed at 250 °C. This enhancement can be attributed to the p-n heterostructure effect. ZIF-67-derived Co_3_O_4_/ZnO heterostructure-based ethanol sensing has also been reported in the literature.^121^ When Co_3_O_4_ integrates with ZnO, the transfer of electrons from ZnO to Co_3_O_4_ and the transfer of holes from Co_3_O_4_ to ZnO occur due to the higher Fermi level of ZnO compared to Co_3_O_4_. This process continues until an equilibrium is established. Under an ambient environment at 250 °C, oxygen molecules are primarily adsorbed as monoionic oxygen species on the surface. It leads to the development of a hole accumulation layer on Co_3_O_4_ and an electron depletion layer (EDL) on the ZnO surface, as illustrated in Figure 14a. When ethanol is injected into the Co_3_O_4_/ZnO heterostructure, the electrons trapped in the adsorbed oxygen ionic species are released back into the conduction band. It reduces the thickness of the accumulation layer on Co_3_O_4_ and the depletion layer on ZnO, as shown in Figure 14b. Therefore, the electrical resistance of the Co_3_O_4_/ZnO heterostructure increases after ethanol injection. Consequently, the Co_3_O_4_/ZnO heterostructure exhibits a higher sensing response to ethanol compared to that of ZnO alone. Thus, the selective detection of ethanol is observed at 250 °C, as shown in Figure 12a. The dynamic electrical resistance changes and gas-sensing response for ethanol exposure in sample 1A at 250 °C are presented in Figure S14­(a,b).

**Figure 14:**
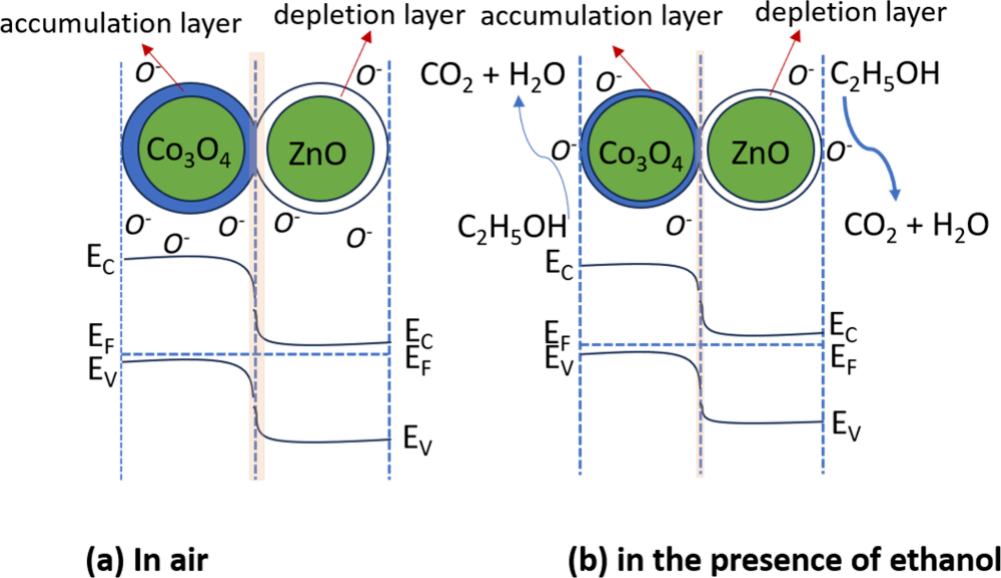
An illustration of energy band diagrams of Co_3_O_4_/ZnO heterostructure: (a) in air and (b) in the presence of ethanol.

In the case of sample M1, the highest sensing responses were observed for n-butanol and 2-propanol at 300 °C (Figure S13­(a)). This may be attributed to Cd doping, which enhances the electron affinity at this temperature, enabling selective detection of these analytes. Previous studies have also reported that Cd doping in ZnO improves n-butanol detection.^114^ The dynamic sensing responses for n-butanol and 2-propanol at 300 °C are shown in Figure S15­(a,b). The response and recovery times calculated for the target gas/analytes with the highest sensing response at corresponding temperatures for all tested samples are summarized in Table S3. The M-series samples, i.e., the samples with Cd doping, exhibit lower response/recovery times compared to the A-series samples. This can be attributed to the larger ionic radius of Cd^2+^ ions (0.78 Å) compared to Zn^2+^ (0.60 Å), which facilitates the formation of more native defects, specifically oxygen vacancies. Additionally, Cd^2+^ acts as a Lewis acid domain to help dissociate oxygen molecules, decreasing the binding energy of adsorbed molecules and leading to faster kinetics.^122^

ZIF-7 is a flexible MOF that undergoes phase transition upon the reversible adsorption of guest molecules. Its pore aperture size can change significantly due to interactions with adsorbates, a phenomenon known as the gate-opening effect. The rotation of benzene rings in the benzimidazole linkers facilitates accommodation of guest molecules, allowing switching between the open phase (5 Å) and closed phase (3 Å) of the ZIF-7 framework.^40^ The diffusion coefficient of guest molecules in ZIF-7 depends on both their kinetic diameter and their interaction with the pore aperture prior to entry. Once equilibrium is established, diffusion remains influenced by molecular size and framework interactions. Hydrogen exhibits the highest diffusion coefficient among the tested molecules due to its small size,^30^ which explains the high selectivity of ZIF-7 toward hydrogen, as shown in Figure 12b.

The more symmetrical state with larger accessible pore volume of ZIF-7 is favorable at higher temperatures, as reported in the literature.^123^ At elevated temperatures (300 °C), sensing responses for n-butanol and ethanol are also observed, likely due to dipolar interactions between the hydrogen atoms of the alcohols and nitrogen atoms in the benzimidazole linkers, as reported in previous studies.^28^ The gas-sensing performance of sample M2 is shown in Figure S13­(b).

For samples 3A and M3, the gas-sensing results indicate that sample M3 exhibits greater selectivity for hydrogen, attributed to the synergistic effect of the Cd doping effect with ZIF-71 coating (Figure 12(c) and Figure S11­(c)). This combination suppresses the responses to other analytes. Studies have also confirmed enhanced hydrogen sensing due to Cd doping.^109,124^

Hydrogen can easily diffuse through ZIF-8 due to its small kinetic diameter (2.89 Å), resulting in a notable sensing response even at lower temperatures, as shown in Figure 12d. Grand Canonical Monte Carlo (GCMC) and molecular dynamics (MD) simulations have shown that due to the hydrophobic nature of ZIF-8, alcohols with longer carbons chains (e.g., n-butanol) are more readily adsorbed.^28^ This is attributed to the abundance of nonpolar aliphatic sites, making n-butanol more favorable for adsorption compared to ethanol or 2-propanol.^28^ Vandezande et al. also reported that apolar molecules like n-butanol are more readily adsorbed on ZIF-8, while 2-propanol adsorption is hindered by its molecular geometry.^125^ Therefore, ZIF-8 exhibits a superior adsorption of n-butanol, resulting in an enhanced sensing performance. The gas-sensing performance for sample M4 is shown in Figure S13­(d).

## 4 Proposed Gas-Sensing Mechanism

The sensing mechanism of the tested analytes on individual sensors can be described as a three-step process: sorption, diffusion, and desorption. The presence of a porous layer of various ZIFs on top of the ZnO surface significantly affects the first step-sorption. These ZIF layers interact with the target gases in different ways depending on their physical and chemical properties, as discussed in the previous section.

The adsorption of gaseous analytes onto the ZIFs alters the electrical properties of the hybrid material.^126^ Subsequent diffusion of the analytes and their interaction at the ZIF/ZnO interface further modulate the electrical characteristics. This combined effect arises from multiple factors: molecular sieving, polarity differences, affinity for functional groups, and gate-opening mechanisms due to aromatic ring rotation, diffusion coefficients, and the resultant electrical changes at the interface between ZIFs and ZnO.

The transport mechanism of target gases through ZIF pores can be understood more efficiently by determining the flow regime of the gases at an operating temperature (*T*) of 300 °C and atmospheric pressure (*p*). To determine the flow regime, the Knudsen number (*K*) can be calculated, which depends on the mean free path (λ) of the test gas, using the following relation:^127,128^

$$\lambda =\frac{k_{B}T}{\surd 2\pi t_{\mathrm{ij}}^{2}p}$$ 10

where *k* *_B_* is the Boltzmann constant and *t*_ij_ is the collision diameter.

The concentration of test gases is 100 ppm, which is negligible compared with background air. The dominance of background air renders the mean free path independent of the small mole fraction of the test gas. Though, the mean free path of the test gas molecule is solely differentiated from the other test gases based on their respective collision diameters.

The mean free path of ethanol, hydrogen, and n-butanol at 300 °C was calculated using eq 10, and the results are 63, 148, and 52 nm, respectively.

The flow regime can be classified into three classes: Knudsen diffusion regime, transitional diffusion regime, and molecular diffusion regime, depending on the Knudsen number (*K*), using the following relation:^127,128^

$$K=\frac{\lambda }{t_{\mathrm{kl}}}$$ 11

where *t* *_kl_* is the pore aperture size of ZIF-67 (*t*_11_), ZIF-7 (*t*_22_), ZIF-71 (*t*_33_), and ZIF-8 (*t*_44_).

The Knudsen numbers of ethanol, hydrogen, and n-butanol are calculated and tabulated below.

The obtained Knudsen numbers for all the test gases and studied ZIFs (Table 5) fall into the Knudsen diffusion regime (*K* > 10). The distribution of ZIF particles form an almost uniform film on the ZnO and Cd-doped ZnO films for all ZIFs, though it is not continuous, as observed in SEM images. Moreover, in all cases, the ZIF layers are not fully free of pinhole defects, and gas transport may also occur through grain boundaries and other defects, which could locally reduce the value of *K* in those regions. It can be concluded that the transport of hydrogen, n-butanol, and ethanol through all the studied ZIFs intrinsically proceeds via Knudsen diffusion. However, contribution from molecular and transitional diffusion may arise locally at grain boundaries and defects.^1,47,128^

**Table 5:** Knudsen Number (*K*) of Tested Gas Molecules for All the Four Studied ZIFs

ZIF names	hydrogen	n-butanol	ethanol
ZIF-67	435	153	185
ZIF-7	493–296	173–104	210–126
ZIF-71	290	102	124
ZIF-8	435	153	185

ZnO, a typical metal oxide semiconductor, exhibits n-type behavior due to the intrinsic defects, which generate donor states just below the conduction band.^129^ When oxygen molecules from the atmosphere are adsorbed onto the ZnO surface, they are transformed into different oxygen ionic species, depending on the operating temperature.^130^ As an oxidizing agent, oxygen accepts electrons, forming an ionic species. In the lower-temperature range, below 100 °C: molecular oxygen ionic species (*O*_2_^*–*^) dominate. Furthermore, in the temperature range between 100 °C < *T* ≤ 300 °C, monoionic oxygen species (O**^–^**) are predominant and bi-ionic oxygen species (O^2–^) become dominant above 300 °C.^130^ These transformations are represented in eqs 12–15.^130^

$$O_{2(\mathrm{gas})}↔O_{2(\mathrm{ads}.)}$$ 12

$$O_{2(\mathrm{ads}.)}+e^{-}↔O_{2(\mathrm{ads}.)}^{-}$$ 13

$$O_{2(\mathrm{ads}.)}+2e^{-}↔2O_{\left(\mathrm{ads}.\right)}^{-}$$ 14

$$O_{\left(\mathrm{ads}.\right)}^{-}+e^{-}↔O_{\left(\mathrm{ads}.\right)}^{2-}$$ 15

where O_2(gas)_, O_2(ads.)_, and *e* ^–^ are the oxygen gaseous molecule in the atmosphere, adsorbed oxygen molecule on the sensing surface, and transferred electron from the conduction band of the ZnO, respectively.

All individual sensors exhibit notable sensing responses within the 150–300 °C range, which highlights the role of monoionic oxygen species (O^–^) in the sensing mechanism. The adsorption of these ionic oxygen species increases the surface work function and induces upward band bending (as shown in Figure 15), resulting in a rise in ZnO electrical resistance.

**Figure 15:**
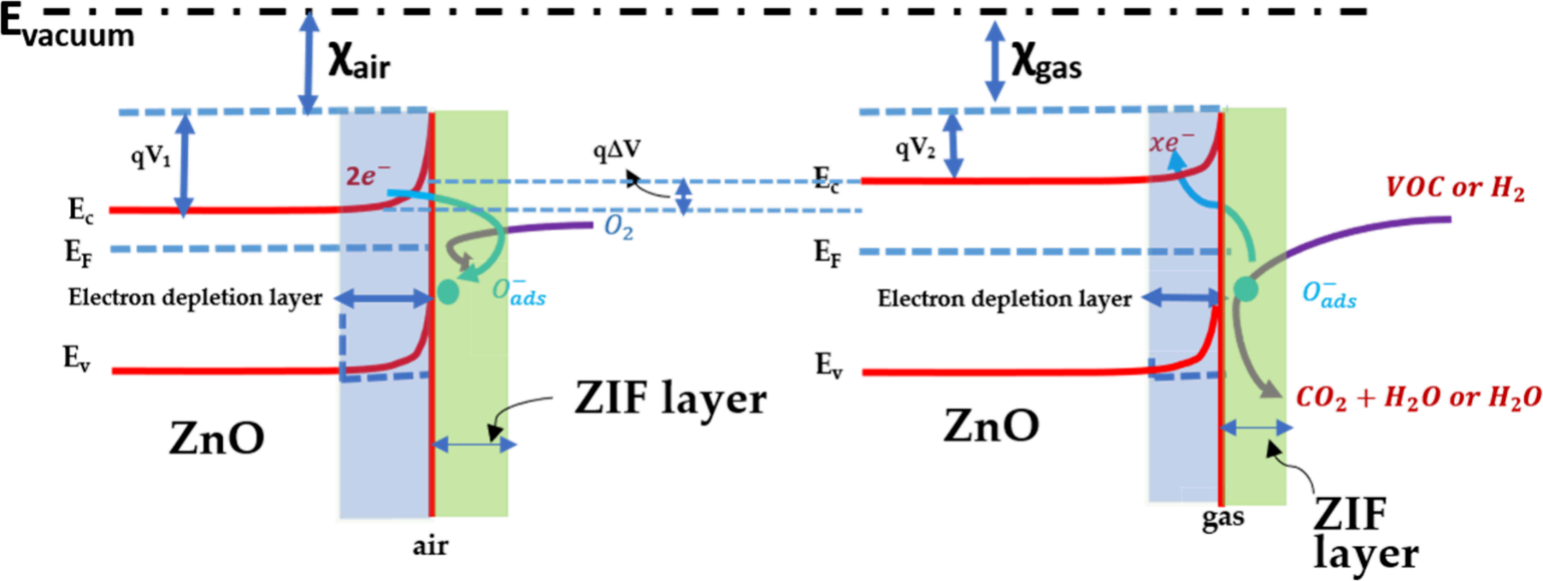
Schematic of the gas-sensing mechanism of ZIF-coated ZnO, illustrating energy band diagrams upon exposure to reducing gases (VOCs and H_2_).

Upon exposure to reducing gases—such as n-butanol, 2-propanol, ethanol, acetone, and hydrogen—the adsorbed gases react with surface-bound oxygen species (O**^–^**), leading to the transfer of electrons back to the conduction band of ZnO, as shown in eqs 16–20.^12,131–133^ This reaction reduces the upward band bending (*q*Δ*V* = *qV*_1_ – *qV*_2_) and leads to a decrease in the electrical resistance. As the electrical resistance decreases, the EDL becomes thinner.^134^

$$(C_{2}H_{5}\mathrm{OH}{)}_{\mathrm{ads}.}+6O_{\mathrm{ads}.}^{-}\to 2{{\mathrm{CO}}_{2}}_{\left(\mathrm{gas}\right)}+3H_{2}O+6e^{-}$$ 16

$$(C_{3}H_{8}O{)}_{\mathrm{ads}.}+9O_{\mathrm{ads}.}^{-}\to 3{{\mathrm{CO}}_{2}}_{\left(\mathrm{gas}\right)}+4H_{2}O+9e^{-}$$ 17

$$(C_{4}H_{9}\mathrm{OH}{)}_{\mathrm{ads}.}+12O_{\mathrm{ads}.}^{-}\to 4{{\mathrm{CO}}_{2}}_{\left(\mathrm{gas}\right)}+5H_{2}O+12e^{-}$$ 18

$$({\mathrm{CH}}_{3}{\mathrm{COCH}}_{3}{)}_{\mathrm{ads}.}+8O_{\mathrm{ads}.}^{-}\to 3{{\mathrm{CO}}_{2}}_{\left(\mathrm{gas}\right)}+3H_{2}O+8e^{-}$$ 19

$$(H_{2}{)}_{\mathrm{ads}.}+O_{\mathrm{ads}.}^{-}\to H_{2}O+e^{-}$$ 20

where (C_2_H_5_OH)_ads_, (C_3_H_8_O)_ads_, (C_4_H_9_OH)_ads_, (CH_3_COCH_3_)_ads_, CO_2(gas)_, H_2(ads)_, and H_2_O are adsorbed ethanol, 2-propanol, n-butanol, acetone, released carbon dioxide, adsorbed hydrogen, and released water, respectively.

## 5 Conclusions

In this study, various ZIFs (ZIF-67, ZIF-7, ZIF-71, and ZIF-8) were successfully deposited onto ZnO and Cd-doped ZnO surfaces, forming MOF/MO_*x*_ hybrid structures via a simple and cost-effective microdrop-casting method. The preferential growth by SCS of ZnO was observed along the *c*-axis corresponding to the (0 0 2) plane. Moreover, the preferential growth for ZIF-67, ZIF-7, ZIF-71, and ZIF-8 particles was found along (0 1 1), (1 1 0), (1 1 0), and (0 1 1) planes, respectively. The crystallite sizes were estimated to be approximately 35.67, 42.30, 61.97, and 37.50 nm for ZIF-67, ZIF-7, ZIF-71, and ZIF-8, respectively. However, the crystallite size calculation for ZIF-71 particles is unreliable in this case due to their large particle size (500 to 700 nm). Additionally, *in situ* XRD analysis of ZIFs from room temperature up to ≥500 °C provided valuable insights into crystal-phase transitions, thermal degradation, and the transformation of ZIFs into their corresponding metal oxides (ZnO or Co_3_O_4_), which were further confirmed through TGA analysis. By increasing the temperature from 30 to 225 °C, the rightward shift of XRD reflection by 0.04 to 0.12 ° was observed, which can be attributed to shrinking of ZIF-67 framework. For ZIF-7, by increasing the temperature from 50 to 100 °C, ZIF-7 transforms from phase-I to phase-II due to solvent extraction. Furthermore, by increasing the temperature from 30 to 350 °C, the shift of XRD reflections was observed to lower angles from 9.47 to 9.04 °, which can be attributed to the thermal expansion of the ZIF-7 framework. Similarly, for ZIF-8, by increasing the temperature from 30 to 325 °C, the rightward shift of XRD reflections by 0.12 ° was observed and the intensity of (0 1 1) reflex decreases due to deterioration of Zn–N bond in the ZIF-8 framework.

Moreover, the morphology and distribution of ZIF particles on ZnO and Cd-doped ZnO surfaces were systematically analyzed. The presence of Cd dopant in ZnO films was confirmed by EDX analysis in Cd-doped ZnO films and supported by the shift in XRD reflections of the M0 sample compared to the 0A sample. In addition, Raman spectroscopy was employed to study the vibrational spectra of all the studied sensors, identifying distinct phonon modes such as methyl bending, N––(Co or Zn)––N stretching, linker stretching and torsional modes (τ), linker bending, and δ-ring deformation. Furthermore, Cd doping in ZnO films caused the 2*A*_1_ (LO) Raman mode peak to shift to higher wavenumbers by 1.68 cm^–1^, which is attributed to increased lattice stress. XPS analysis revealed the surface chemistry of studied ZIFs on the ZnO surface, namely, MOF/MO_*x*_ hybrid structures. It provided significant information about the intactness of the ZIFs on the sample surface. Moreover, it also provides insight into the extent of degradation within the ZIF-71 framework in the presence of X-rays due to the presence of specific functional sites (−Cl). It provides a critical understanding of the radiolytic stability of halogenated MOFs by identifying X-ray-induced degradation in ZIF-71. It elucidates how specific functional groups dictate structural integrity under ionizing radiation and defining the thresholds necessary for nondestructive characterization. During current–voltage characteristics, inverted hysteresis was observed by employing forward and reverse voltage sweep, which can be characterized as capacitive hysteresis attributed to charge traps that slow down the return path.

Furthermore, sequential gas-sensing experiments demonstrated notable selectivity and sensitivity to ethanol (notably with sample 1A), 2-propanol and n-butanol (with samples M1, 1A, 4A, M2, and M4), and hydrogen (with samples 2A, 3A, 4A, M2, M3, and M4). By Cd doping in ZnO, the selectivity for MOF/MO_*x*_ hybrid structures to n-butanol and hydrogen was observed at 150 and 300 °C, respectively. The transport of the test gases intrinsically proceeds via Knudsen diffusion through ZIFs. In all cases, the sensor showed good repeatability, underscoring the robustness and reliability of the developed sensors. Future research will focus on long-term stability tests to determine the temporal robustness of the developed sensors. Further optimization of the ZIF layer thickness and device-level integration and testing could enhance the practical sensing performance. This work expands the scope of radiolytic detection and effects by identifying X-ray-induced degradation within the ZIF framework. Specifically, it elucidates how functional groups dictate structural integrity under ionizing radiation and defines the thresholds required for nondestructive characterization. Finally, this work proposes a predictive framework for the selective detection of hydrogen and VOCs through the strategic selection of dopants and ZIF topologies, utilizing MOF/MO_*x*_ hybrid architectures for environmental and biomedical applications.

## Supplementary Material



## Notes

Schematic of the device structure of the ZIF particles on a ZnO or ZnO:Cd film. SEM micrographs of ZnO- and Cd-doped ZnO films; XRD pattern of sample 2A (ZIF-7-coated ZnO), highlighting the characteristic XRD peaks of ZIF-7; XRD patterns of ZIF-67, ZIF-7, and ZIF-71 on Si substrate during *in situ* heating experiment; Raman spectra of 0A sample and M0 sample; Raman spectra of ZIF-coated Cd-doped ZnO samples; zoom-in Raman spectra of 1A and M1 samples to show the peak shift due to Cd doping; elemental composition of samples M1–M4 obtained by semiquantitative XPS analysis; comparison of N 1s, O 1s, C 1s, and Cl 2p XPS high-resolution spectra of the M3 sample from this study and a ZIF-71-coated Al-doped CuO film from a previous study after respective charge correction and fitting; absolute *E*_*b*_ positioning of all fitting functions used in the XPS analysis; O 1s XPS high-resolution spectra of the M1, M2, M3, and M4 sample; I–V characteristics of 0A and M0 samples at different temperatures; I–V characteristics of M1, M2, M3, and M4 samples at different temperatures; gas-sensing results of different gases for 0A and M0 samples, at different operating temperatures in the range of 20 to 300 °C; gas-sensing results of different gases for M1, M2, M3, and M4 samples, at different operating temperatures in the range of 20 to 300 °C; dynamic electrical resistance changes and dynamic gas-sensing response of sample 1A in the presence of ethanol at 250 °C; dynamic gas-sensing response of sample M1 in the presence of n-butanol and 2-propanol at 300 °C; response and recovery times calculated for the target gas/analytes (PDF)
