# Supplementary material for: In Situ Temperature-Dependent Properties of Metal–Organic Framework ZIF-Coated ZnO Hybrid Structures: Structural and Advanced Spectroscopic Insights
Source: ACS Omega. 2026 May 4;11(19):29178–201. doi: 10.1021/acsomega.6c02238 (PMC13191689; doi:10.1021/acsomega.6c02238)
Supplement: Supplementary file 1 [file ao6c02238_si_001.pdf]

Supporting information

In-situ temperature-dependent properties of metal-organic frameworks ZIF-coated ZnO hybrid structures: structural and advanced spectroscopic insights

Rajat Nagpal [1,2], Masaya Sugihara [3], Cristian Lupan [1], Nicolae Magariu [1], Tim Tjardts [4], Nahomy Meling Lizarde [4], Thomas Strunskus [4,6], Justin Jetter [5], Haoyi Qiu [2], Barnika Chakraborty [2], Tayebeh Ameri [4,6], Eckhard Quandt [5], Rainer Adelung [2,6,], Rob Ameloot [3], Oleg Lupan [1*]*

[1] Center for Nanotechnology and Nanosensors, Department of Microelectronics and Biomedical Engineering, Faculty CIM, Technical University of Moldova, 168 Stefan cel Mare str., MD-2004, Chisinau, Republic of Moldova

[2] Chair for Functional Nanomaterials, Department of Materials Science, Faculty of Engineering, Kiel University, Kaiserstraße 2, D-24143 Kiel, Germany

[3] Centre for membrane separations, Adsorption, Catalysis, and Spectroscopy (cMACS), KU Leuven, 3001 Leuven, Belgium

[4] Chair for Composite Materials, Department of Materials Science, Faculty of Engineering, Kiel University, Kaiserstraße 2, D-24143 Kiel, Germany

[5] Chair of Inorganic Functional Nanomaterials, Department of Material Science, Faculty of Engineering, Kiel University, Kaiserstraße 2, D-24143 Kiel, Germany

[6] Kiel Nano, Surface and Interface Science KiNSIS, Kiel University, Christian Albrechts-Platz 4, 24118, Kiel, Germany

*Corresponding authors:
Rainer Adelung (ra@tf.uni-kiel.de)
Oleg Lupan (ollu@tf.uni-kiel.de, oleg.lupan@mib.utm.md)

KEYWORDS: MOFs; ZIFs; in-situ; defects; hybrid materials; sequential detection; sensor mechanism

Schematic of device structure

The development process of the ZIF-coated metal oxide-based hybrids is consistent with previously reported literature [1–4].

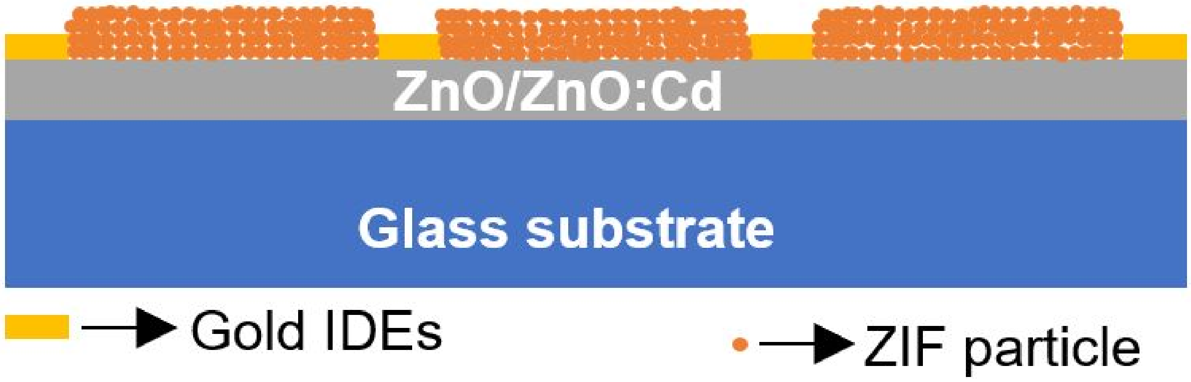

Figure S1. Schematic of the device structure: ZIF particles on a ZnO or ZnO:Cd films, supported by a glass substrate with gold IDEs for electrical contacts.

Compositional and morphological properties

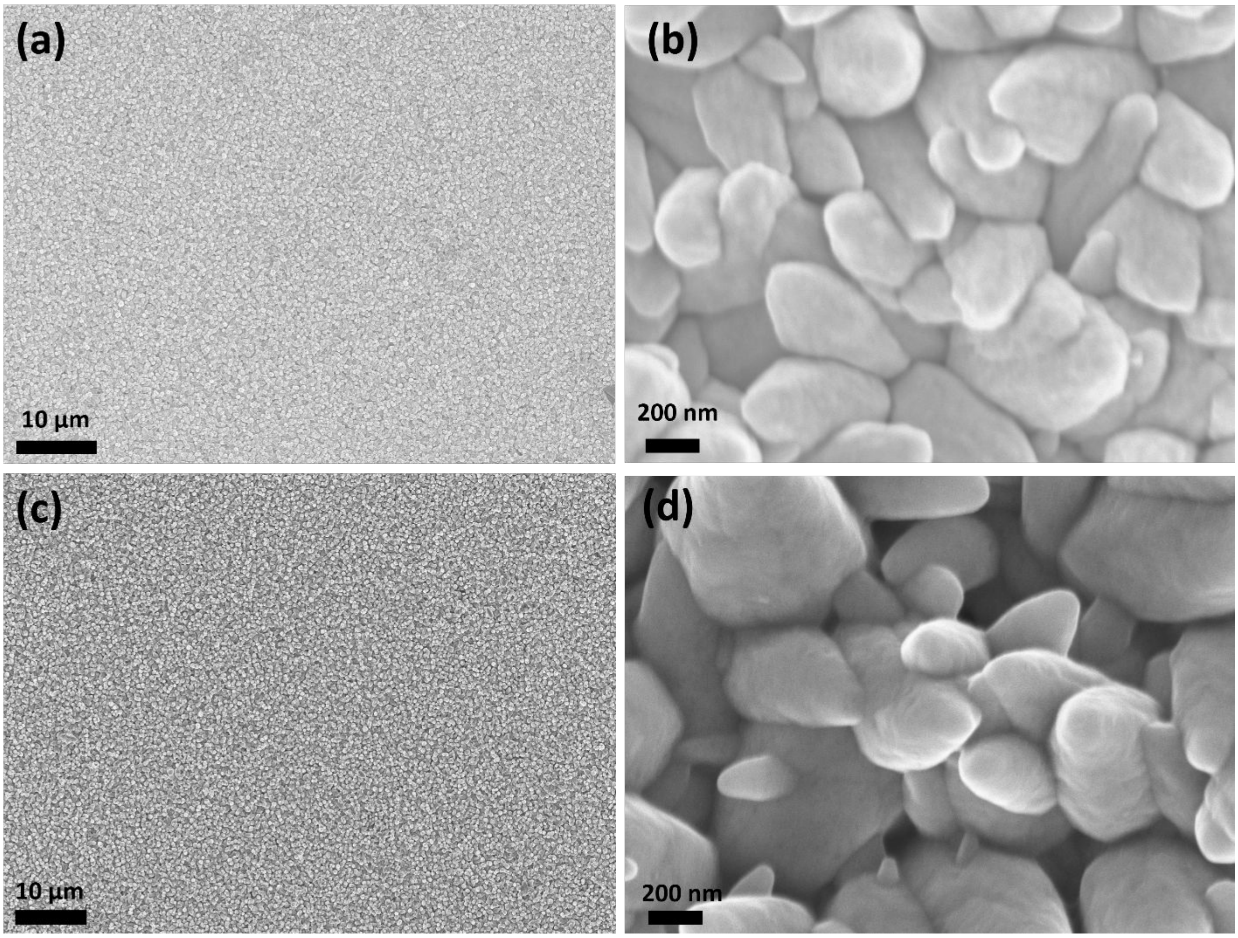

Figure S2. SEM micrographs of the ZnO and Cd-doped ZnO films at lower magnifications (a) and (c); and higher magnifications (b) and (d), respectively.

Structural properties

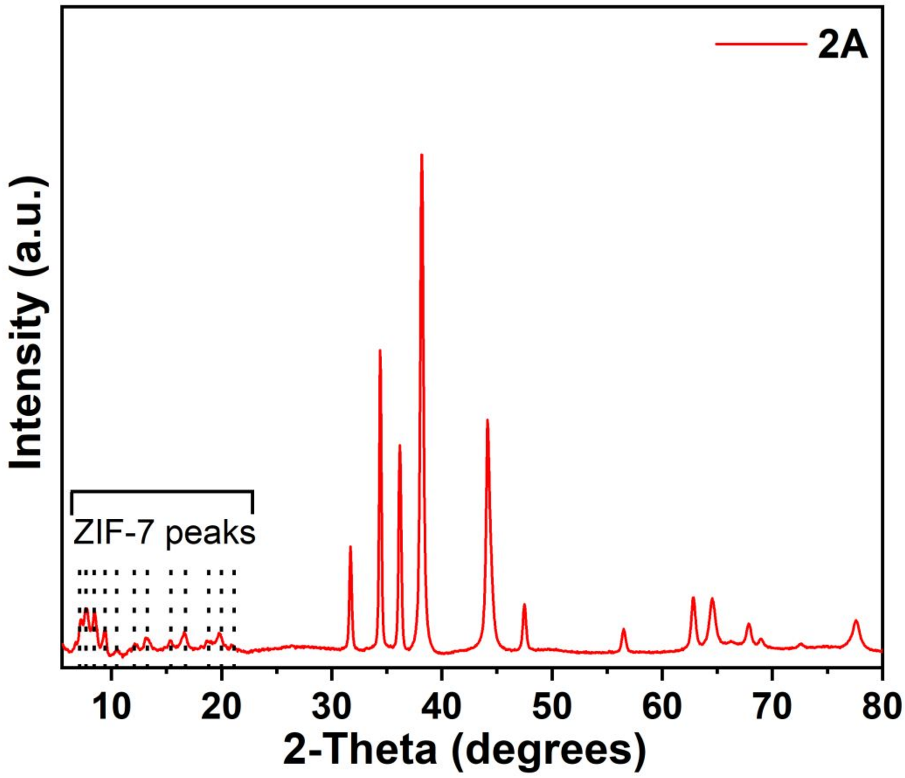

Figure S3. XRD pattern of sample set 2A (ZIF-7-coated ZnO), highlighting the characteristic XRD peaks of ZIF-7.

In-situ structural properties

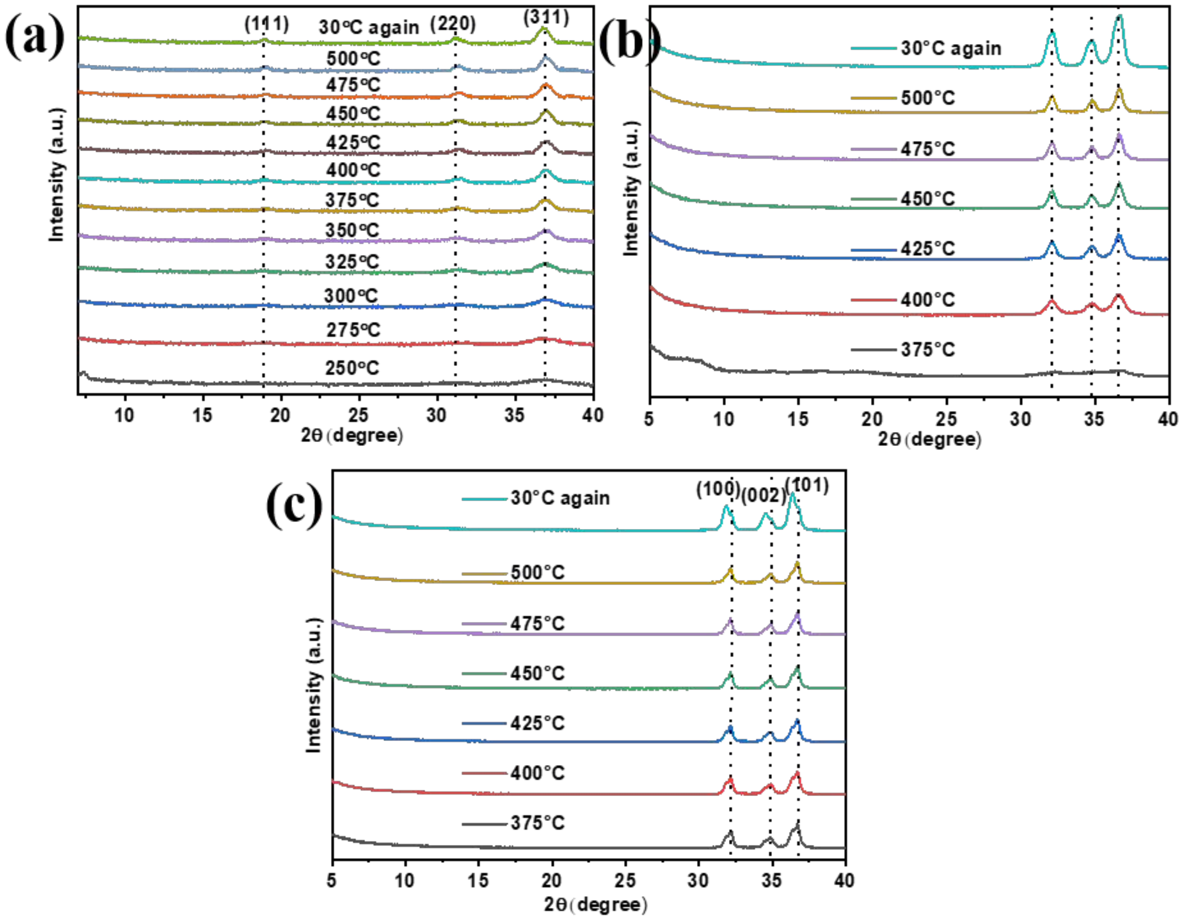

Figure S4. XRD patterns of (a) ZIF-67; (b) ZIF-7, and (c) ZIF-71 on Si substrate during *in-situ* heating experiments.

Vibrational properties

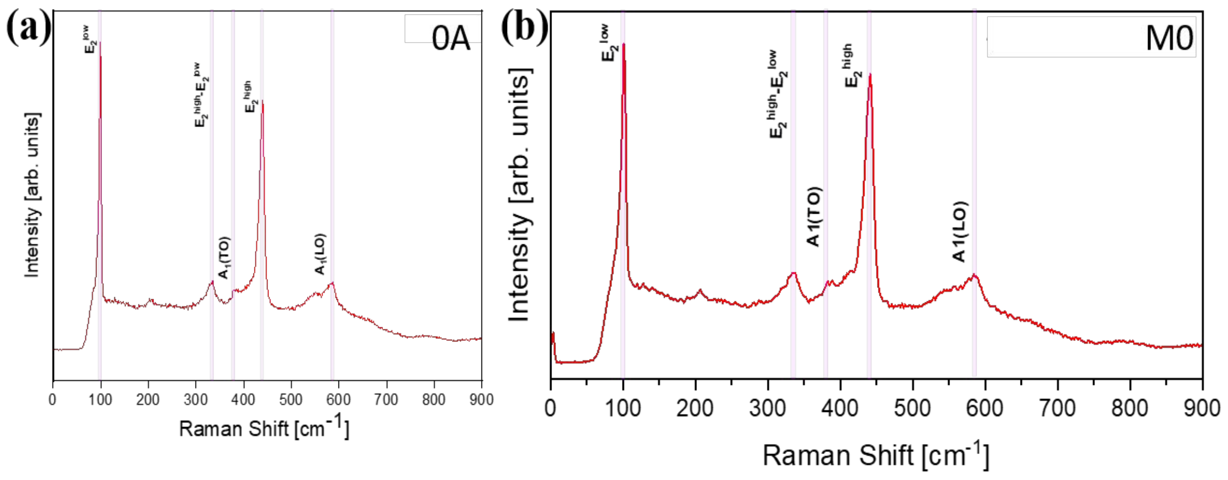

Figure S5. (a) Raman spectra of the 0A (ZnO) sample set, and (b) M0 (Cd-doped ZnO) sample set.

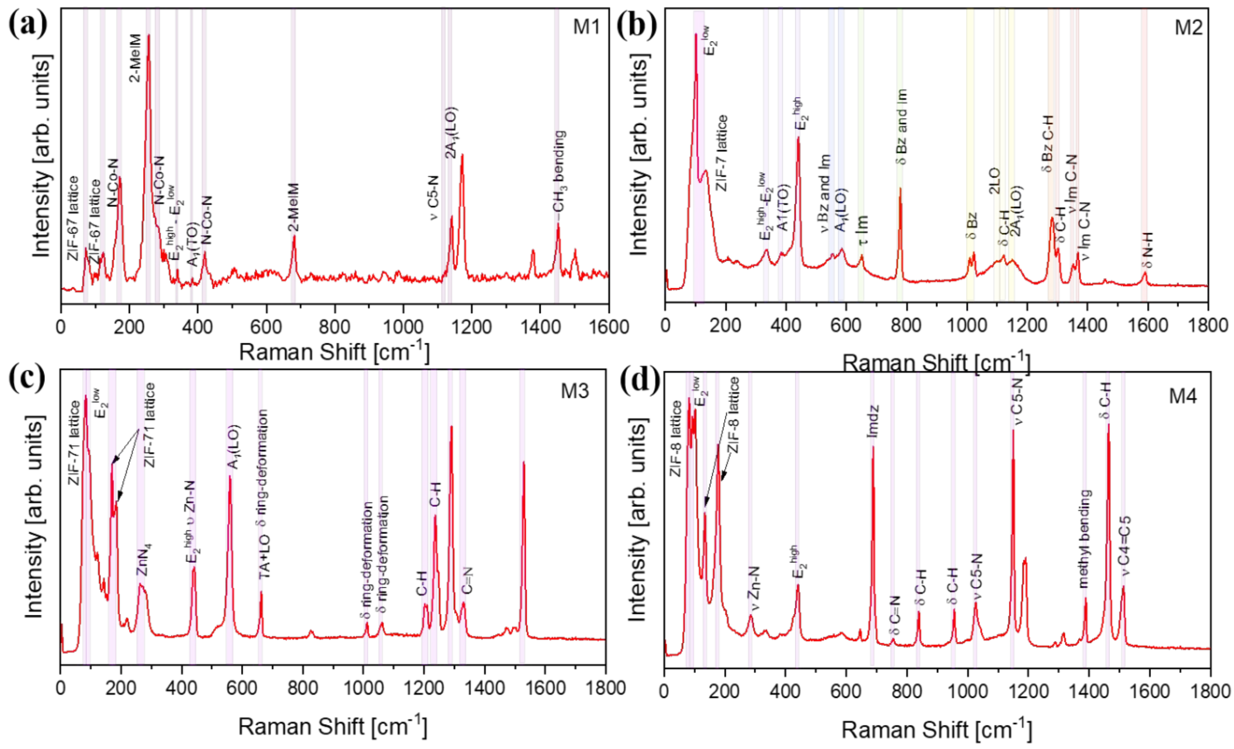

Figure S6. Raman spectra of the ZIF-coated Cd-doped ZnO samples: (a) M1 (ZIF-67-coated Cd-doped ZnO); (b) M2 (ZIF-7-coated Cd-doped ZnO); (c) M3 (ZIF-71-coated Cd-doped ZnO); and (d) M4 (ZIF-8-coated Cd-doped ZnO) sample sets.

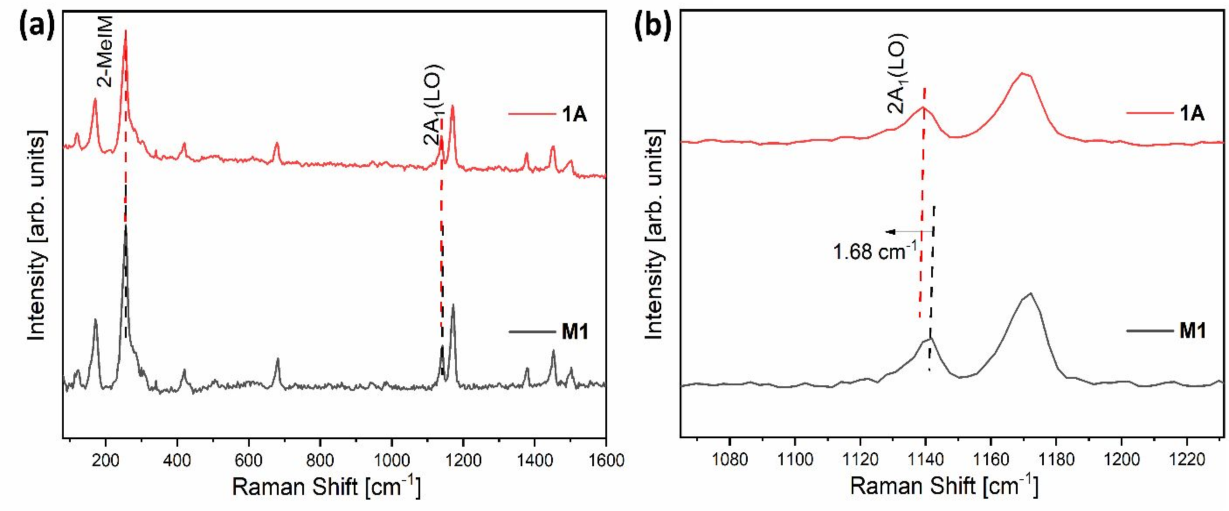

Figure S7. Raman spectra of (a) 1A (ZIF-67-coated ZnO) and M1 (ZIF-67-coated Cd-doped ZnO) samples, (b) zoom-in spectra of (a) to show the peak shift due to Cd doping.

Chemical properties using XPS

Table S1. Elemental composition of samples M1–M4 obtained by semiquantitative XPS analysis.

**Element Composition (at%)**	**C** - C 1s	**O** - O 1s	**N** - N 1s	**Zn** - Zn 2p	**Cl** - Cl 2p	**Co** - Co 2p
M1 (ZIF-67)	64.8	4.0	24.8	0.6	-	5.9
M2 (ZIF-7)	72.1	2.6	18.6	6.7	-	-
M3 (ZIF-71)	49.6	1.8	19.4	6.2	22.4	-
M4 (ZIF-8)	61.1	3.1	26.2	9.5	-	-

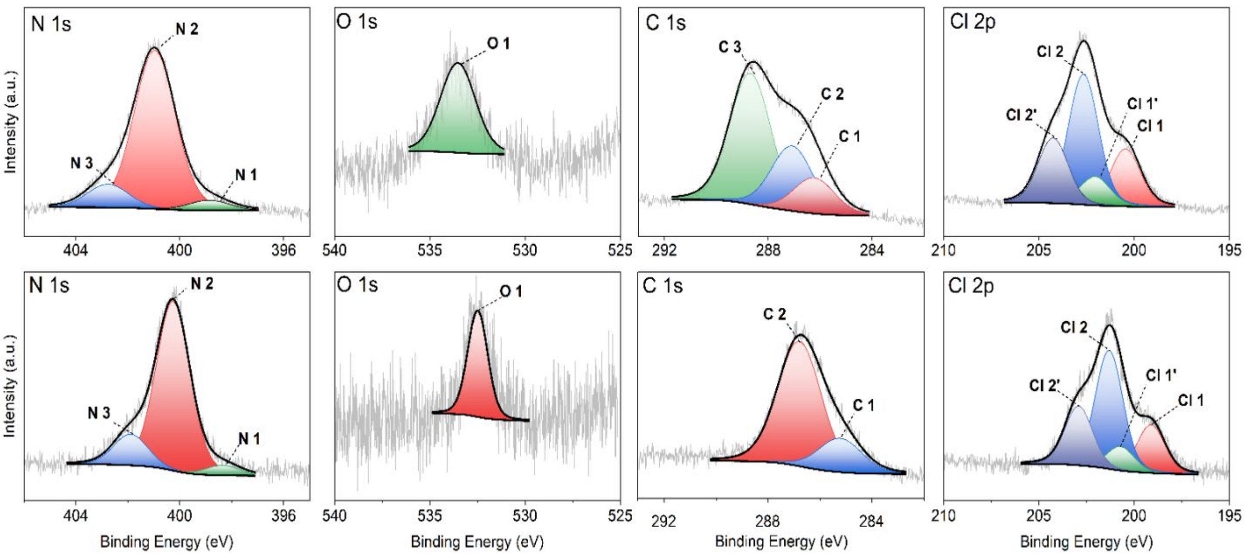

Figure S8. Comparison of N 1s, O 1s, C 1s, and Cl 2p XPS high-resolution spectra of the M3 (ZIF-71-coated Cd-doped ZnO) sample from this study and a ZIF-71 coated Al-doped CuO film from a previous study[2] after respective charge correction and fitting.

Table S2. Absolute E_b positions of all fitting functions used in the XPS analysis presented in the main manuscript Figure 7 and supporting information Figure S7.

**Element**	**Components**	**Binding Energy (eV)**
	**M1**	
C 1s	C 1	286.7
Co 2p	Co 1	781.2
Co 2p	Co 1'	796.7
Co 2p	Satellites	786.4
Co 2p	Satellites	802.4
N 1s	N 1	400.3
O 1s	O 1	532.3
Zn 2p	Zn 1	1020.5

Element	Components	Binding Energy (eV)
Zn 2p	Zn 1'	1043.5
**M2**		
C 1s	C 1	286.2
C 1s	C 2	287.3
N 1s	N 1	400.6
O 1s	O 1	533.0
Zn 2p	Zn 1	1021.7
Zn 2p	Zn 1'	1044.6
**M3**		
C 1s	C 3	288.7
C 1s	C 2	287.1
C 1s	C 1	286.2
Cl 2p	Cl 1	200.4
Cl 2p	Cl 2	202.7
Cl 2p	Cl 1'	202.0
Cl 2p	Cl 2'	204.3
N 1s	N 3	402.8
N 1s	N 2	401.0
N 1s	N 1	398.9
O 1s	O 1	533.5
Zn 2p	Zn 1	1021.7
Zn 2p	Zn 1'	1044.6
**M4**		
C 1s	C 1	286.9
N 1s	N 2	400.6
N 1s	N 1	398.7
O 1s	O 1s	532.9
Zn 2p	Zn 2p 3/2	1021.7
Zn 2p	Zn 2p 1/2	1044.7

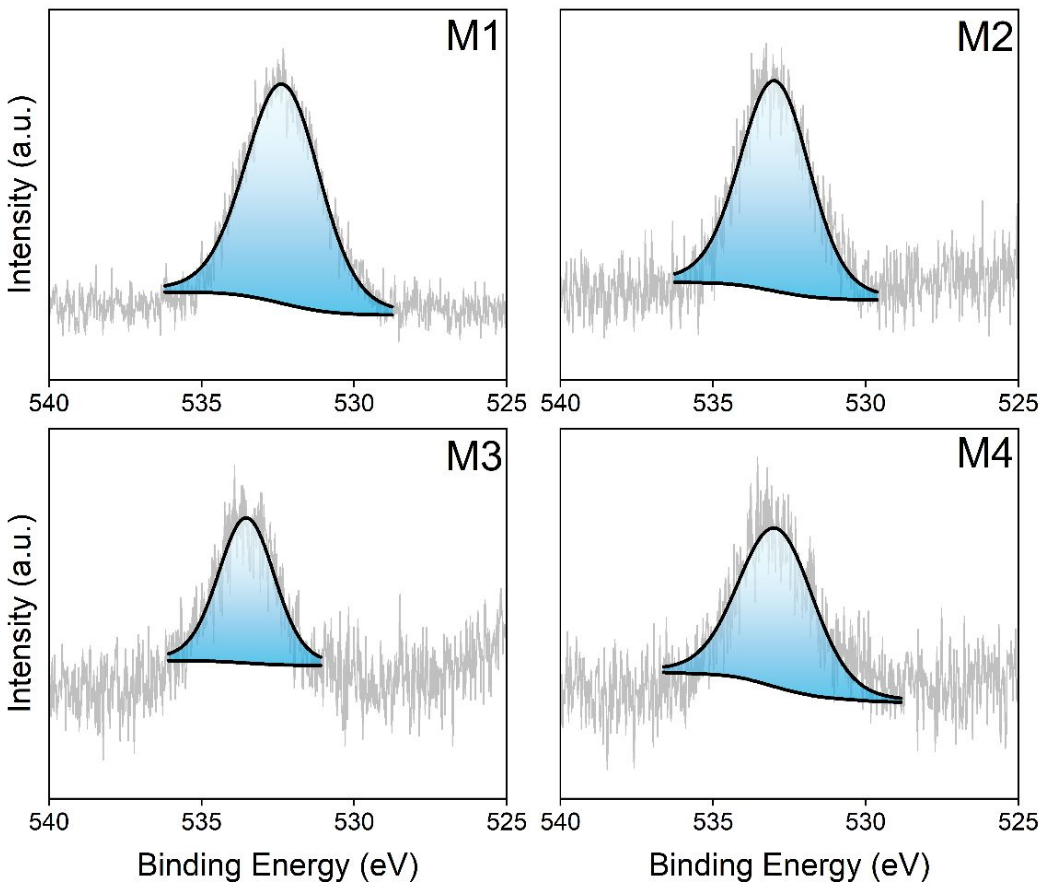

Figure S9. O-1s XPS high-resolution spectra of the M1 (ZIF-67-coated Cd-doped ZnO), M2 (ZIF-7-coated Cd-doped ZnO), M3 (ZIF-71-coated Cd-doped ZnO), and M4 (ZIF-8-coated Cd-doped ZnO) samples.

Electrical properties

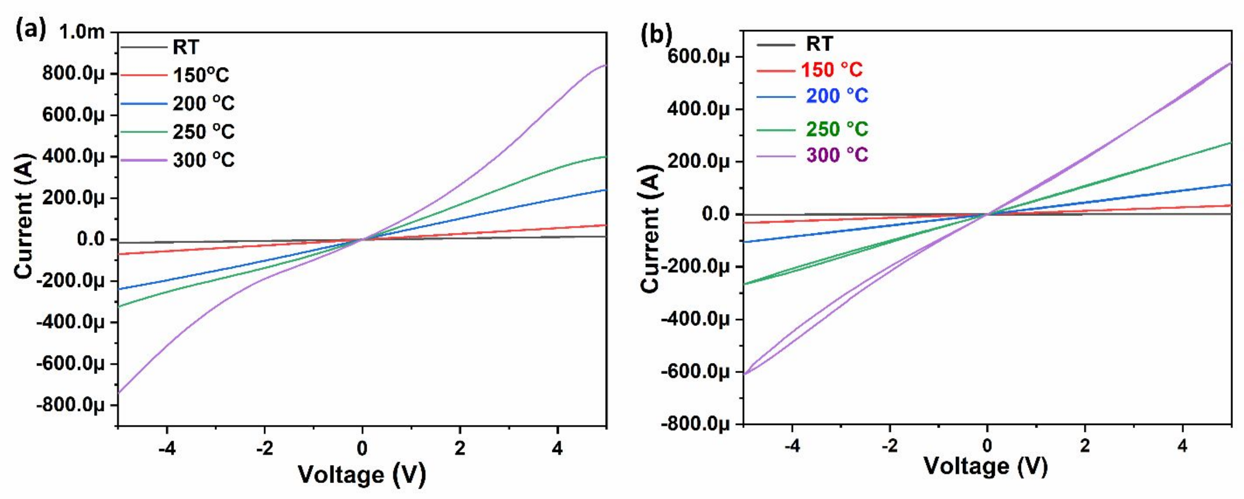

Figure S10. Current-Voltage I-V characteristics of: (a) 0A (ZnO) and (b) M0 (Cd-doped ZnO) samples at different temperatures.

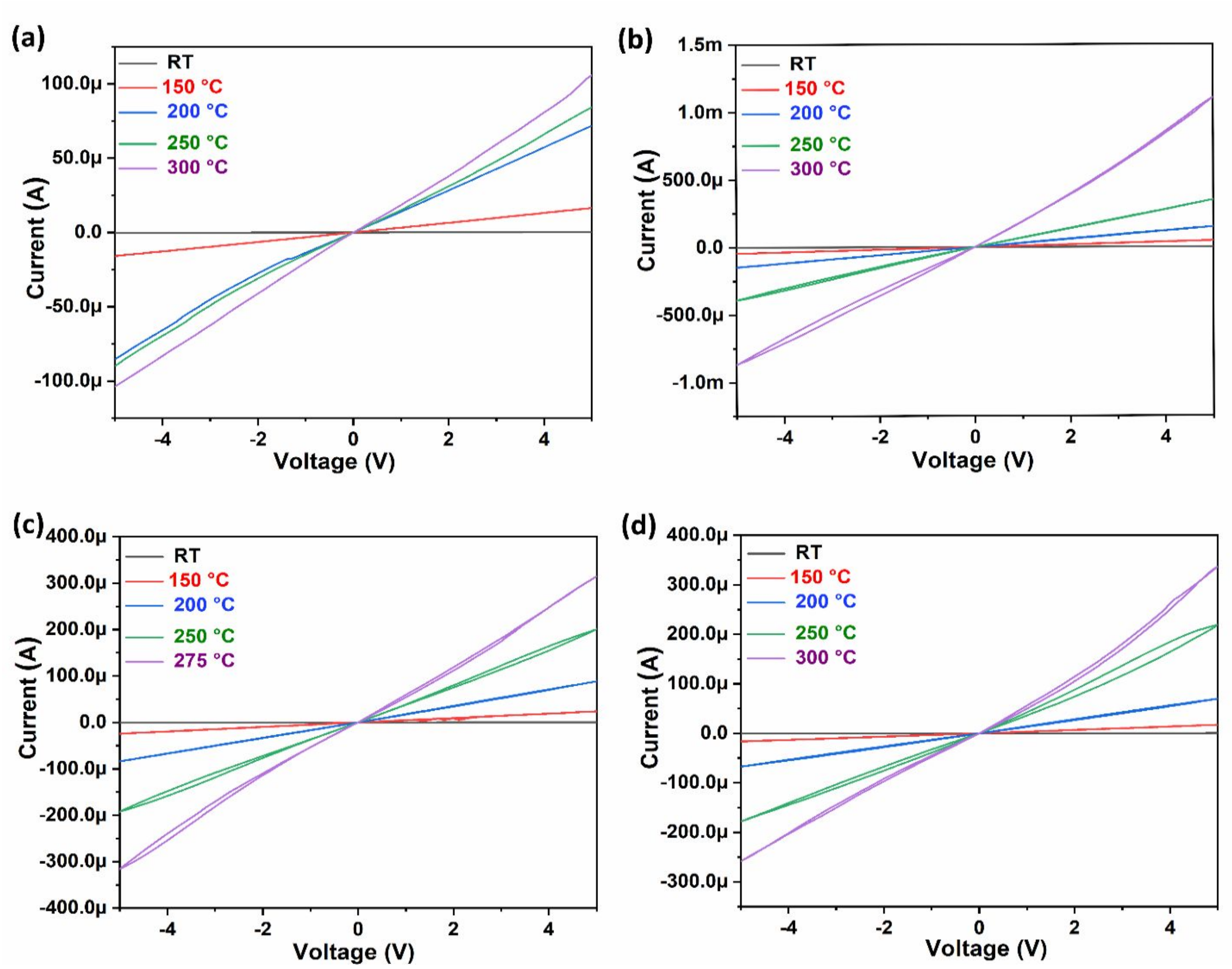

Figure S11. Current-Voltage I-V characteristics of (a) M1 (ZIF-67-coated Cd-doped ZnO), (b) M2 (ZIF-7-coated Cd-doped ZnO), (c) M3 (ZIF-71-coated Cd-doped ZnO), and (d) M4 (ZIF-8-coated Cd-doped ZnO) samples at different temperatures.

Sensing properties

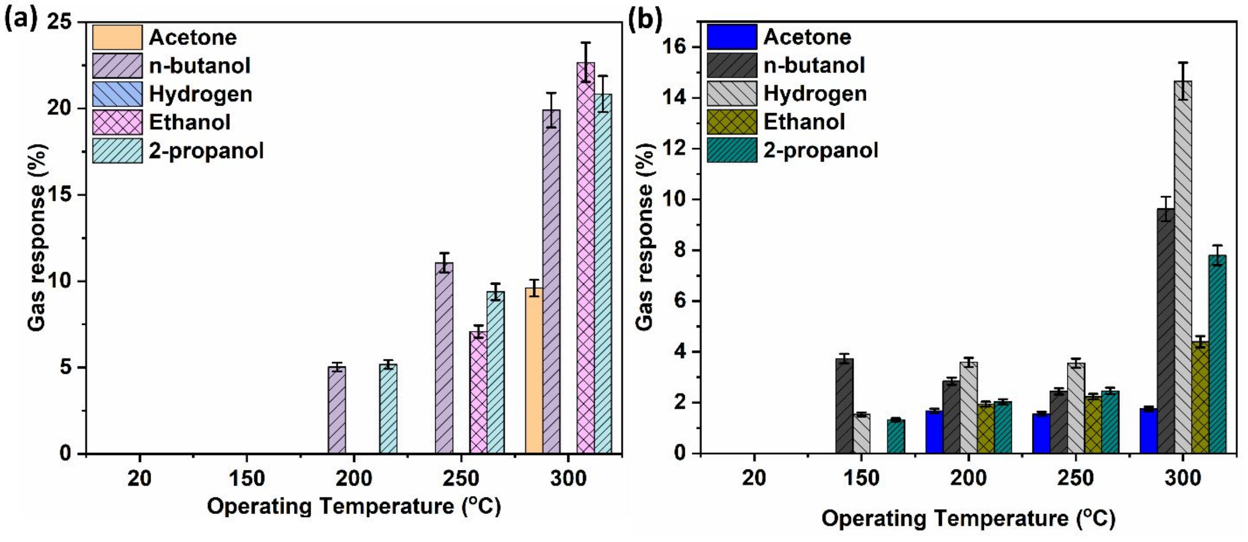

Figure S12. Gas sensing results for different gases for (a) 0A (ZnO) and (b) M0 (Cd-doped ZnO) samples, at different operating temperatures in the range of 20 to 300℃.

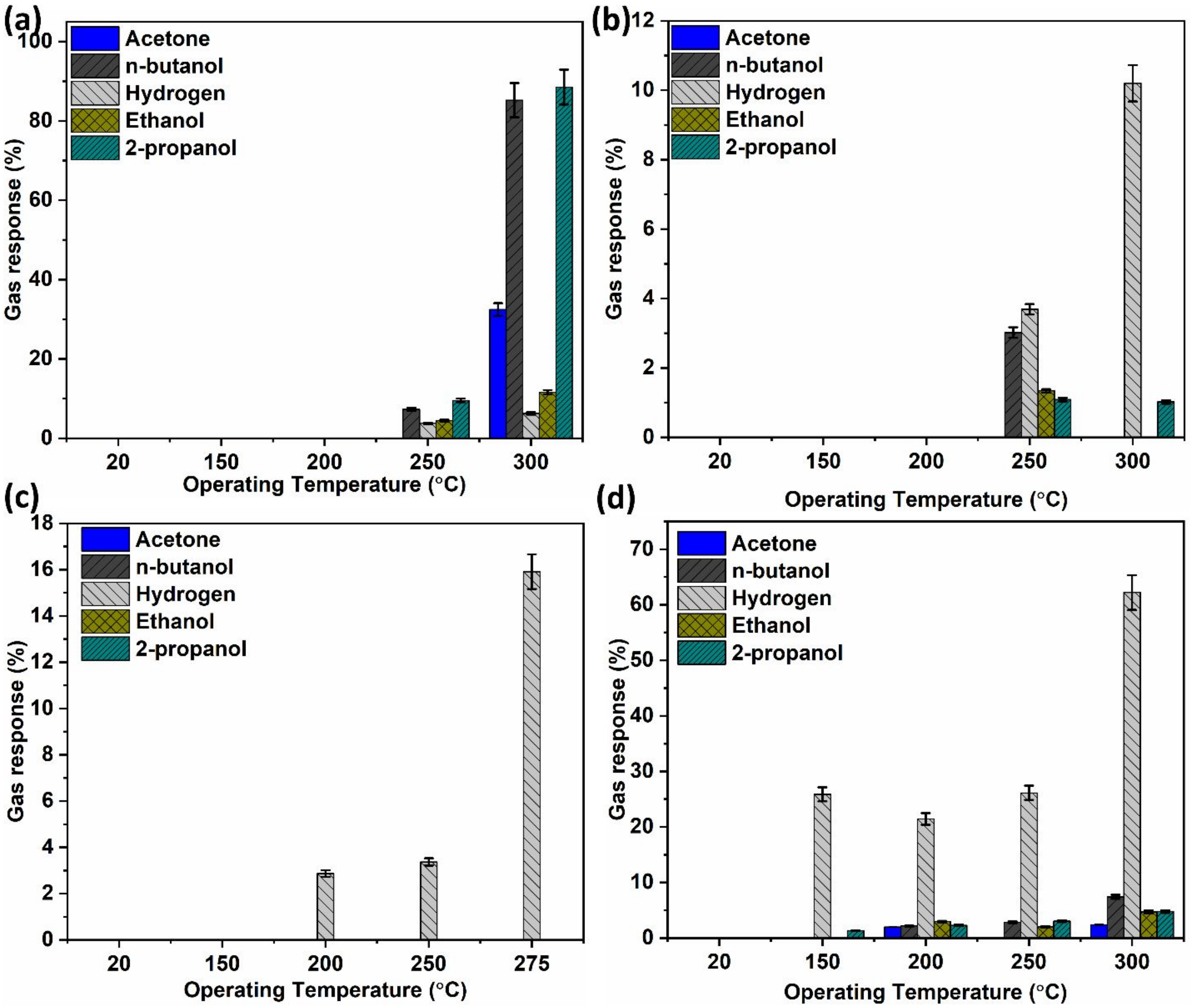

Figure S13. Gas sensing results of different gases for: (a) M1 (ZIF-67-coated Cd-doped ZnO), (b) M2 (ZIF-7-coated Cd-doped ZnO), (c) M3 (ZIF-71-coated Cd-doped ZnO), and (d) M4 (ZIF-8-coated Cd-doped ZnO) samples, at different operating temperatures in the range of 20 to 300℃.

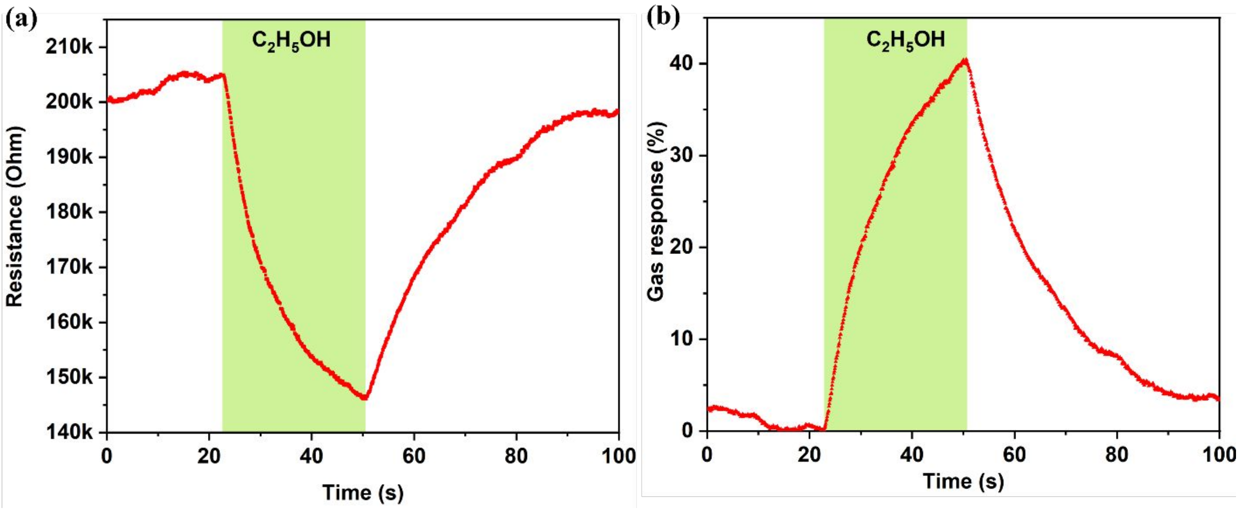

Figure S14. (a) Dynamic resistance changes and (b) Dynamic gas sensing response of the sample 1A set in the presence of ethanol at 250°C.

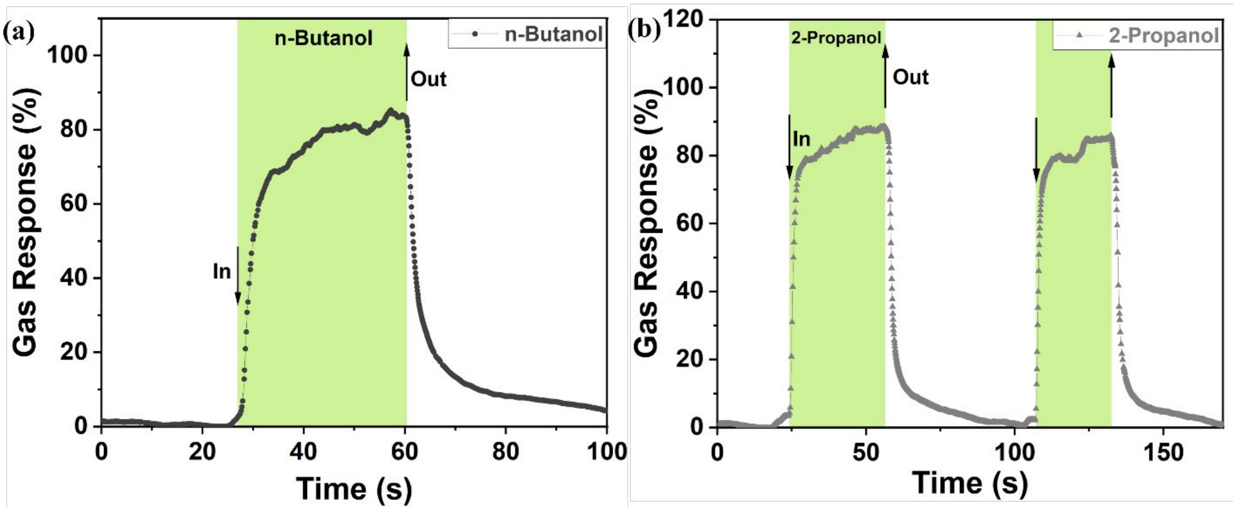

Figure S15. Dynamic gas sensing response of the sample M1 set (ZIF-67-coated Cd-doped ZnO) in the presence of: (a) n-butanol and (b) 2-propanol at 300°C.

Table S3: Response and recovery times calculated for the target gas/analytes with the highest sensing response at corresponding temperatures for all tested samples.

Sample name	Test gases/ analytes	Operating temperature, ℃	Response time, s	Recovery time, s
1A (ZIF-67 coated ZnO)	Ethanol	250	19	36
1A (ZIF-67 coated ZnO)	2-propanol	250	16	15
1A (ZIF-67 coated ZnO)	n-butanol	250	20	37
1A (ZIF-67 coated ZnO)	Hydrogen	250	11	9
2A (ZIF-7 coated ZnO)	Hydrogen	250	8	29
2A (ZIF-7 coated ZnO)	Hydrogen	300	13	33
3A (ZIF-71 coated ZnO)	Hydrogen	250	13	28
3A (ZIF-71 coated ZnO)	2-propanol	250	14	17
3A (ZIF-71 coated ZnO)	n-butanol	250	8	6
3A (ZIF-71 coated ZnO)	Ethanol	250	5	3
4A (ZIF-8 coated ZnO)	Hydrogen	250	14	41
4A (ZIF-8 coated ZnO)	Hydrogen	300	19	26
M1 (ZIF-67 coated Cd-doped ZnO)	n-butanol	250	20	22
M1 (ZIF-67 coated Cd-doped ZnO)	Ethanol	250	16	17
M1 (ZIF-67 coated Cd-doped ZnO)	2-propanol	250	2	9
M1 (ZIF-67 coated Cd-doped ZnO)	2-propanol	300	4	9
M2 (ZIF-7 coated Cd-doped ZnO)	Hydrogen	250	2	19
M2 (ZIF-7 coated Cd-doped ZnO)	Hydrogen	300	2	10
M3 (ZIF-71 coated Cd-doped ZnO)	Hydrogen	250	6	14
M3 (ZIF-71 coated Cd-doped ZnO)	Hydrogen	275	8	12
M4 (ZIF-8 coated Cd-doped ZnO)	Hydrogen	250	2	8
M4 (ZIF-8 coated Cd-doped ZnO)	Hydrogen	300	8	13

References

(1) Nagpal, Rajat; Sugihara, M.; Magariu, N.; Tjardts, T.; Meling-Lizarde, N.; Strunskus, T.; Ameri, T.; Ameloot, R.; Adelung, R.; Lupan, O. Humidity-Tolerant Selective Sensing of Hydrogen and n-Butanol Using ZIF-8 Coated CuO:Al Film. *Mater. Chem. Front.* **2025**, *9* (23), 3425–3442. https://doi.org/10.1039/D5QM00565E.

(2) Nagpal, Rajat; Sugihara, M.; Lupan, C.; Tjardts, T.; Meling-Lizarde, N.; Strunskus, T.; Qiu, H.; Adelung, R.; Ameloot, R.; Lupan, O. ZIF-71-Coated CuO:Al with Enhanced Gas-Sensing Performance for n-Butanol and Hydrogen. *ACS Appl. Electron. Mater.* **2025**, *7* (22), 10198–10215. https://doi.org/10.1021/acsaelm.5c01659.

(3) Lupan, O.; Nagpal, Rajat; Litra, D.; Brinza, M.; Sugihara, M.; Ameloot, R.; Railean, S.; Ameri, T.; Adelung, R.; Schröder, S.; Faupel, F. Hybrid Nanomaterials for Biomedical Sensors. In *7th International Conference on Nanotechnologies and Biomedical Engineering*; Sontea, V., Tiginyanu, I., Railean, S., Eds.; Springer Nature Switzerland: Cham, 2025; pp 162–176; DOI: 10.1007/978-3-032-06494-3_18.

(4) Nagpal, Rajat; Ababii, N.; Lupan, O. Comprehensive Advances in Gas Sensing: Mechanisms, Material Innovations, and Applications in Environmental and Health Monitoring. *Materials Today Electronics* **2025**, vol. 15, 100192. https://doi.org/https://doi.org/10.1016/j.mtelec.2025.100192.
